# Early antenatal care initiation and associated factors in Hohoe Municipality, Ghana: A five-year multi-facility repeated cross-sectional record review study

**DOI:** 10.1371/journal.pone.0356427

**Published:** 2026-11-04

**Authors:** Marina Sewornu, Dickson Kweku Delali, Hubert Amu, Richard Gyan Aboagye, Francis Bruno Zotor

**Affiliations:** 1 Department of Family and Community Health, Fred N. Binka School of Public Health, University of Health and Allied Sciences, Hohoe, Ghana; 2 Department of Epidemiology and Biostatistics, Fred N. Binka School of Public Health, University of Health and Allied Sciences, Hohoe, Ghana; 3 Department of Theoretical and Applied Biology, Kwame Nkrumah University of Science and Technology, Kumasi, Ghana; 4 Department of Population and Behavioural Sciences, Fred N. Binka School of Public Health, University of Health and Allied Sciences, Hohoe, Ghana; PLOS: Public Library of Science, UNITED KINGDOM OF GREAT BRITAIN AND NORTHERN IRELAND

## Abstract

**Background:**

Early initiation of antenatal care (ANC) is a critical strategy for improving maternal and neonatal health outcomes, as it enables timely detection and management of pregnancy-related risks. However, women in low- and middle-income countries initiate ANC late. This study estimated the proportion of women initiating ANC within the first trimester in three health facilities in the Hohoe Municipality, Ghana, over a five-year period (2020-2024). It also assessed trends across years and examined factors associated with early ANC initiation.

**Methods:**

We conducted a multi-facility repeated cross-sectional record review of ANC records from three health facilities in Hohoe Municipality from January 2020 to December 2024. Of 7,660 records identified, 1,381 were excluded because the first ANC contact had occurred at another facility, leaving 6,279 for analysis. Background characteristics were summarised using frequencies and percentages, and the proportion of women initiating ANC within the first trimester was calculated overall and for each year. Multivariable binary logistic regression was used to examine the factors associated with early ANC initiation; age-by-parity interaction terms tested, and facility-by-predictor interaction models were fitted to assess effect heterogeneity. Results were presented as odds ratios with 95% confidence intervals (CIs), and statistical significance was set at p < 0.05.

**Results:**

Early ANC initiation increased slightly from 45.3% in 2020 to 46.4% in 2023 before declining to 44.3% in 2024, with an overall proportion of 45.1% across the study period. Patterns differed by facility between the COVID-19 (2020–2021) and post-COVID-19 (2022–2024) periods. Early initiation declined at Gbi-Wegbe Health Center (54.3% to 46.0%; p = 0.008) but increased at Adabraka Health Center (31.5% to 42.0%; p < 0.001) and at the Volta Regional Hospital (45.4% to 47.6%; p = 0.002). The odds of early ANC initiation was higher among women aged 31–40 years (aOR = 1.15; 95% CI: 1.01–1.31) than those aged 14-30, and among women with high-end of the acceptable range blood pressure (aOR = 1.44; 95% CI: 1.26–1.65), and those with above acceptable/Grade 1 hypertension (aOR = 1.95; 95% CI: 1.34–2.83) relative to those with acceptable hypertension status. Odds were lower with increasing parity, and lower at Adabraka Health Center and Volta Regional Hospital than at Gbi-Wedbe Health Center. The interaction analysis between maternal age and parity showed that multiparous women aged 31–40 had lower odds of early ANC initiation (OR = 0.61; 95% CI: 0.41–0.91), and lower among grand multiparous women of the same age (OR = 0.29; 95% CI: 0.11–0.77). In the facility-stratified analyses, grand multiparity was the only factor associated with early ANC initiation, which was consistent across all three facilities.

**Conclusion:**

Fewer than half of women initiated ANC early in the Hohoe Municipality, with less variations across the study period and divergent trajectories between facilities. Early initiation was more likely among women aged 31–40 years and those with raised blood pressure, and less likely with increasing parity, with age and parity interacting. Facility-specific interventions prioritising women with high parity and reproductive education and needed to increase ANC initiation, whiles improving access to health facilities.

## Background

Maternal mortality remains a critical global challenge. In 2023, pregnancy-related complications caused an estimated 260,000 deaths globally, translating to approximately 712 deaths each day and one maternal death every two minutes [1,2]. The global maternal mortality ratio stands at 197 deaths per 100,000 live births, representing a 40% decline since 2000 [1]. However, the pace of this decline has slowed considerably since 2016, and 70% of all maternal deaths now occur in sub-Saharan Africa, with Central and Southern Asia contributing nearly 17% [1,3]. Early antenatal care (ANC) remains one of the most effective strategies for interrupting this pattern, enabling timely identification of hypertension, anaemia, infections, and fetal growth concerns before they escalate, while delivering iron supplementation, tetanus immunisation, HIV screening, and malaria prevention [4]. In response to mounting evidence, the World Health Organization (WHO) replaced the four-visit focused model in 2016 with an eight-contact schedule that explicitly requires the first contact to occur by 12 weeks of gestation [4].

Despite this guidance, timely initiation remains a persistent global challenge. Evidence shows that early ANC coverage in developing regions stood at only 48.1% as of 2013, compared with 84.8% in developed regions, with coverage as low as 24.0% in low-income countries [5]. While approximately 85% of women in developed regions initiate care in the first trimester, only 48% do so in developing regions, and this figure falls to just 25% in sub-Saharan Africa [5]. Pooled analyses of Demographic and Health Survey (DHS) data confirm that only 36.9% of pregnant women across the region start antenatal care in the first trimester, while late initiation exceeds 53.8% [6,7]. Rural residence, low education, higher parity, poverty, and distance to facilities consistently delay first visits, even where services are provided free of charge [8–10].

Ghana has made measurable progress within this broader regional context. The National Health Insurance Scheme removed direct fees for antenatal care, and the 2022 Ghana DHS reports that 63% of women initiated care in the first trimester, with secondary analyses placing nationwide early initiation rates at 65–66% [11–13]. Urban residence, higher education, and lower parity increase the likelihood of early initiation, while rural residence and higher parity reduce it [8,13,14]. Despite these gains, completion of all eight contacts remains low, at 38–44%; regional disparities persist; and the COVID-19 pandemic reversed much of this progress [15,16]. ANC visits declined by nearly 22,000 per month on average across more than 500 Ghanaian facilities during restriction periods, driven by movement limitations and fear of infection, with rural districts recording the slowest recovery [17–19].

Within the Volta Region, Jasikan District, which shares borders, demographics, and health infrastructure with Hohoe Municipality, reports delayed initiation in 45.7% of women, attributed to long waiting times, cultural beliefs, distance, and negative perceptions of early care-seeking [20]. Yet, Hohoe Municipality itself remains understudied, and existing evidence has three critical gaps. First, national surveys and DHS analyses capture point-in-time snapshots but cannot document how the COVID-19 disruptions of 2020–2024 shaped the annual trajectory of early initiation at the local level. Second, no facility-level study exists for Hohoe, making it impossible to identify disparities in ANC timing across care levels from primary health centers to a regional referral hospital within a single catchment area. Third, DHS instruments do not routinely capture clinical variables documented in facility registers, including blood pressure classification and parity, all of which this study demonstrates are independent determinants of when women seek care. This study addresses all three gaps by determining the prevalence, trends, and determinants of early ANC initiation using five years of routine facility records from Hohoe Municipality, providing actionable evidence for the Municipal Health Directorate and contributing to Ghana’s progress toward SDG 3 by 2030.

## Methods

### Study design

A multi-facility repeated cross-sectional record review was conducted using ANC records from three health facilities in Hohoe Municipality from January 2020 to December 2024. Data were extracted between 1 October and 26 December 2025, focusing on documented cases of pregnant women who accessed care within the selected facilities. Record-review designs are widely used in maternal health research to analyse routinely collected hospital data and identify determinants of maternal and neonatal outcomes [21,22]. Cross-sectional analytical approaches are also commonly employed to estimate the prevalence of maternal health service utilisation and examine associated factors within a defined population [23]. The Strengthening the Reporting of Observational Studies in Epidemiology (STROBE) guideline was used in writing the paper (S1 File) [24].

### Study site

Hohoe Municipality is located in the Volta Region of Ghana and covers approximately 1,172 km^2^, with a total population of 114,472 according to the 2021 Population and Housing Census [25]. The municipality has a mixed rural-urban character, with agriculture as the predominant economic activity. It has twenty-one health facilities, including a regional referral hospital, spanning primary to tertiary levels of care [26].

### Study population

The study population comprised pregnant women who attended ANC services in Gbi-Wegbe Health Center, Adabraka Health Center, and Volta Regional Hospital within Hohoe Municipality between January 2020 and December 2024. Data were obtained from routinely collected ANC registers and maternal health records in the three facilities.

### Inclusion and exclusion criteria

Records were eligible for inclusion if they documented antenatal care initiated between January 2020 and December 2024 at Gbi-Wegbe Health Center, Adabraka Health Center, and Volta Regional Hospital in Hohoe Municipality. Eligible records were required to contain complete information on gestational age or the date of the first antenatal care visit, which was essential for classifying ANC initiation as early or late in accordance with WHO recommendations [4]. Completeness of additional key variables, including maternal age and parity, was also required for inclusion in the determinants analysis.

Records were excluded if they belonged to women who had transferred from other facilities, as indicated by a transfer inscription on their records, since their initial antenatal care was initiated outside the study facilities and therefore did not reflect the ANC initiation patterns of the study settings. Records falling outside the study period of January 2020 to December 2024 were also excluded. Data validation procedures embedded in the electronic extraction tool prevented duplicate entries before final submission.

### Sample size determination

Given that this study involved a multi-facility repeated cross-sectional record review of ANC records, a census approach was employed. The denominator comprised all women who made their first ANC visit at the selected facilities during the study period; repeat visits and subsequent ANC contacts by the same woman were excluded. All eligible first-visit records from January 2020 to December 2024 were included across the three facilities: Gbi-Wegbe Health Center (n = 1,408), Adabraka Health Center (n = 1,647), and Volta Regional Hospital (n = 3,224), yielding a total of 6,279 records.

### Study variables

#### Outcome variable:

The outcome variable was early ANC initiation, defined as the first ANC visit taking place at or before 12 weeks of gestation (first trimester). This binary outcome aligns with the WHO 2016 recommendation for a positive pregnancy experience, which recommends initial contact by 12 weeks as part of an eight contact model [4]. The gestational age at first visit was recorded categorically and recoded as binary: early initiation (≤12 weeks) was coded as 1 (yes), and late initiation (>12 weeks) as 0 (no).

#### Exposure variables:

The exposure variables included age, parity, hypertension status, and health facility of attendance. These variables were selected based on their clinical relevance, consistent association with early ANC initiation in the existing literature, and availability in routine facility records throughout the study period. We categorised maternal age as 13–30, 31–40, and 41–49 years. We classified parity as nullipara, primipara, multipara, and grand multipara. Blood pressure categories followed World Health Organization/International Society of Hypertension (WHO/ISH) aligned thresholds: acceptable (<120/ < 80 mmHg); high end of acceptable (120–139 and/or 80–89 mmHg); above acceptable/Grade 1 (140–159 and/or 90–99 mmHg); moderately high/Grade 2 (160–179 and/or 100–109 mmHg); and high/Grade 3 (≥180 and/or ≥110 mmHg) [27]. However, due to data distribution limitations, we combined moderately high/Grade 2 and high/Grade 3 categories into a single category in our study. The three health facilities used for the study were coded as 1 = Gbi-Wegbe Health Center, 2 = Adabraka Health Center, and 3 = Volta Regional Hospital.

### Sampling and data collection procedure

The study used a census approach, reviewing all eligible ANC records from Gbi-Wegbe Health Center, Adabraka Health Center, and Volta Regional Hospital from January 2020 to December 2024. We purposively selected these three facilities to capture different levels of healthcare delivery within Hohoe Municipality. Gbi-Wegbe Health Center and Adabraka Health Center were chosen to represent primary care in rural and peri-urban communities, where most women first access ANC. The Volta Regional Hospital was included to reflect referral-level care, where more complex cases are managed, and ANC data tend to be more comprehensive. Together, these three facilities represent the major providers of ANC services within Hohoe Municipality. Including every facility in the municipality would have spread resources too thin, introduced variability in record-keeping quality, and risked compromising data accuracy. Focusing on facilities with well-documented registers and high patient flow allowed the study to generate robust findings that capture diverse care settings and ANC patterns across the municipality.

Within each facility, all ANC records that met the inclusion criteria were reviewed. Extracted information included maternal socio-demographic characteristics, such as age; clinical and obstetric characteristics, including parity, and blood pressure; and ANC details, such as gestational age at the first ANC visit. Data were collected using a structured extraction sheet developed specifically for this study, which enabled systematic entry, efficient data cleaning, and management of the large dataset. Trained research assistants abstracted the data, while the principal investigator carefully checked all entries for completeness and accuracy before analysis. Conducting a census of all eligible records maximised statistical power, improved representativeness, and reduced selection bias, aligning with recommended practices for a multi-facility repeated cross-sectional record review study. We initially identified 7,660 records across the three facilities. Of these, 1,381 records were excluded on the basis of transfer inscription: 325 at Gbi-Wegbe Health Center, 274 at Adabraka Health Center, and 782 at Volta Regional Hospital, as these women had initiated ANC at another facility prior to attending the study facilities, leaving 6,279 records for the final analysis. The flow of records from identification through to the final analysed sample is illustrated in Fig 1.

**Fig 1:**
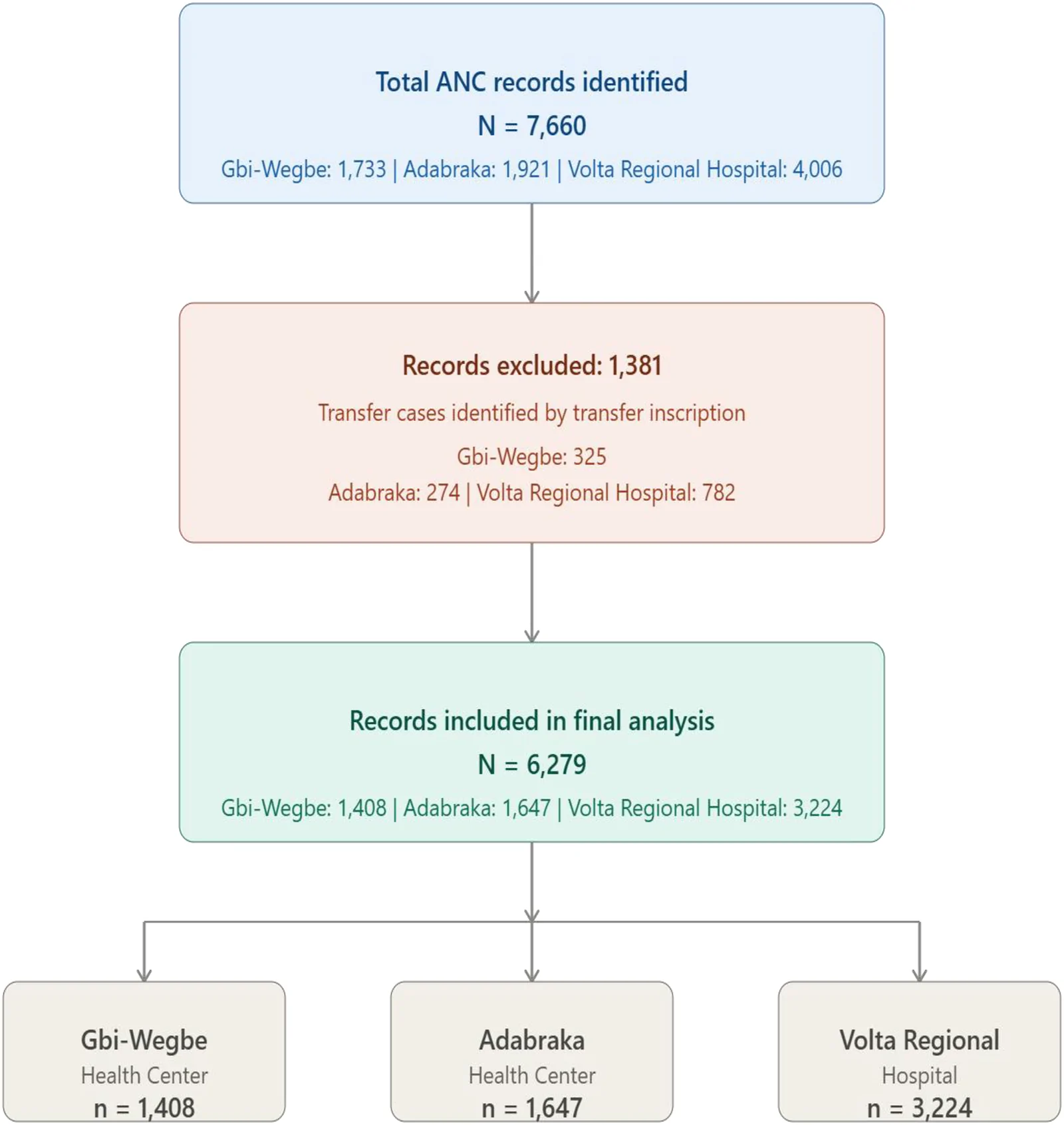
Flow diagram showing the identification, extraction, and inclusion of ANC records from three health facilities in Hohoe Municipality, Ghana, 2020–2024.

### Validity

To ensure validity, the study used data directly from official antenatal care registers and maternal health records maintained at the selected facilities. These registers are standardised across Ghanaian health facilities and are routinely updated by trained healthcare professionals, thereby improving the accuracy and authenticity of the recorded information. The data extraction sheet was aligned with the variables documented in the registers, ensuring that all relevant data were captured consistently. Additionally, the extraction sheet was pretested on a small subset of records not included in the final analysis to assess clarity, completeness, and alignment with the study objectives, thereby enhancing content validity. Implausible values, defined as gestational age outside the range of 4–42 weeks, were flagged and excluded. Data quality was further enhanced through a source document verification process, whereby a second researcher cross-checked all entered data against the original ANC registers before final submission, ensuring the accuracy of data transferred from source records to the electronic extraction tool.

### Data analysis

The data were analysed using Stata version 17.0 (StataCorp, College Station, TX, USA). Frequencies and percentages were used to summarise the background characteristics of the women, and the mean and standard deviation of age were computed to describe the study population. Trend analysis was conducted to examine the prevalence of early ANC initiation from 2020 to 2024, with results presented as percentages. To formally assess the temporal trend, logistic regression models were fitted with survey year included as both a continuous and categorical predictor. Crude odds ratios (CORs) were estimated using binary logistic regression to assess the unadjusted association between each exposure variable and early ANC initiation, followed by adjusted odds ratios (AORs) in a multivariable model that included all covariates regardless of bivariate significance. Statistical significance was set at p < 0.05 with 95% confidence intervals (CI). Interaction terms between maternal age and parity were tested to examine whether the effect of age on early ANC initiation differed across parity categories. Facility-stratified analyses were conducted by fitting separate logistic regression models for each facility to explore whether determinants of early ANC initiation differed across settings. Heterogeneity was formally assessed using pooled logistic regression models that included facility-by-predictor interaction terms, and, separately, period-by-facility interaction terms to test whether the difference between the COVID-19 and post-COVID-19 periods varied by facility. Predictive margins were estimated to illustrate the practical magnitude of each significant predictor across all covariate categories.

### Ethics approval

Ethical approval for this study was obtained from the University of Health and Allied Sciences, Fred N. Binka School of Public Health Ethics Review Committee under Protocol Identification Number UHAS/FNBSPH/PG/{052}25–26 (S2 File). Approval was also obtained from all three health facilities (Gbi-Wegbe Health Center, Adabraka Health Center, and Volta Regional Hospital) before the commencement of this study. All data were handled confidentially, and no personally identifiable information was recorded. The study complied with the principles of the Declaration of Helsinki for research involving human data [28].

## Results

### Background characteristics of pregnant women

In Table 1, we present the background characteristics of the respondents. The age ranged from 14 to 49 years, with a mean of 27.6 ± 0.08 years. More than half of the women were aged 14–30 (66.8%). The majority had acceptable blood pressure readings (80.5%). Regarding facility attendance, most women received care at Volta Regional Hospital (51.4%). From the pooled prevalence of early ANC initiation among the women from 2020 to 2024, it was estimated that 2834 (45.1%) of the women initiated ANC early from all three health facilities included in the study.

**Table 1:** Background characteristics of pregnant women.

Variable	Frequency (n = 6279)	Percentage (%)
**Age (mean age = 27.6 ± 0.08)**		
14-30	4192	66.8
31-40	1948	31.0
41-49	139	2.2
**Parity**		
Nullipara	1964	31.3
Primipara	1631	26.0
Multipara	2445	38.9
Grand Multipara	239	3.8
**Hypertension status (mmHg)**		
Acceptable	5051	80.5
High end of acceptable	1089	17.3
Above acceptable/Grade 1	120	1.9
Moderately high/Grade 2 & High Grade 3	19	0.3
**Facility**		
Gbi-Wegbe Health Center	1408	22.4
Adabraka Health Center	1647	26.2
Volta Regional Hospital	3224	51.4
**ANC initiation**		
Early	2834	45.1
Late	3445	54.9

### Trends in early ANC initiation by year (2020–2024)

Fig 2 presents the trends in early ANC initiation from 2020 to 2024. Across the five years, less than half of the women initiated ANC early. In 2020, 45.3% of women initiated ANC early, which declined marginally to 44.7% in 2022. The prevalence rose steadily to 46.4% in 2023 and declined to 44.3% in 2024. To formally assess whether a temporal trend existed, logistic regression models were fitted with survey year as a predictor. When entered as a continuous variable, survey year was not a statistically significant predictor of early ANC initiation (OR = 1.00; 95% CI: 0.96–1.03; p = 0.926). When entered as a categorical variable with 2020 as the reference year, no individual year differed significantly from 2020 in the odds of early initiation (2021: OR = 0.98, p = 0.781; 2022: OR = 0.97, p = 0.739; 2023: OR = 1.04, p = 0.577; 2024: OR = 0.96, p = 0.607). After adjusting for age, parity, hypertension status, and facility of attendance, survey year remained non-significant (OR = 1.02; 95% CI: 0.99–1.06; p = 0.207), confirming the absence of a meaningful overall temporal trend in early ANC initiation over the study period.

**Fig 2:**
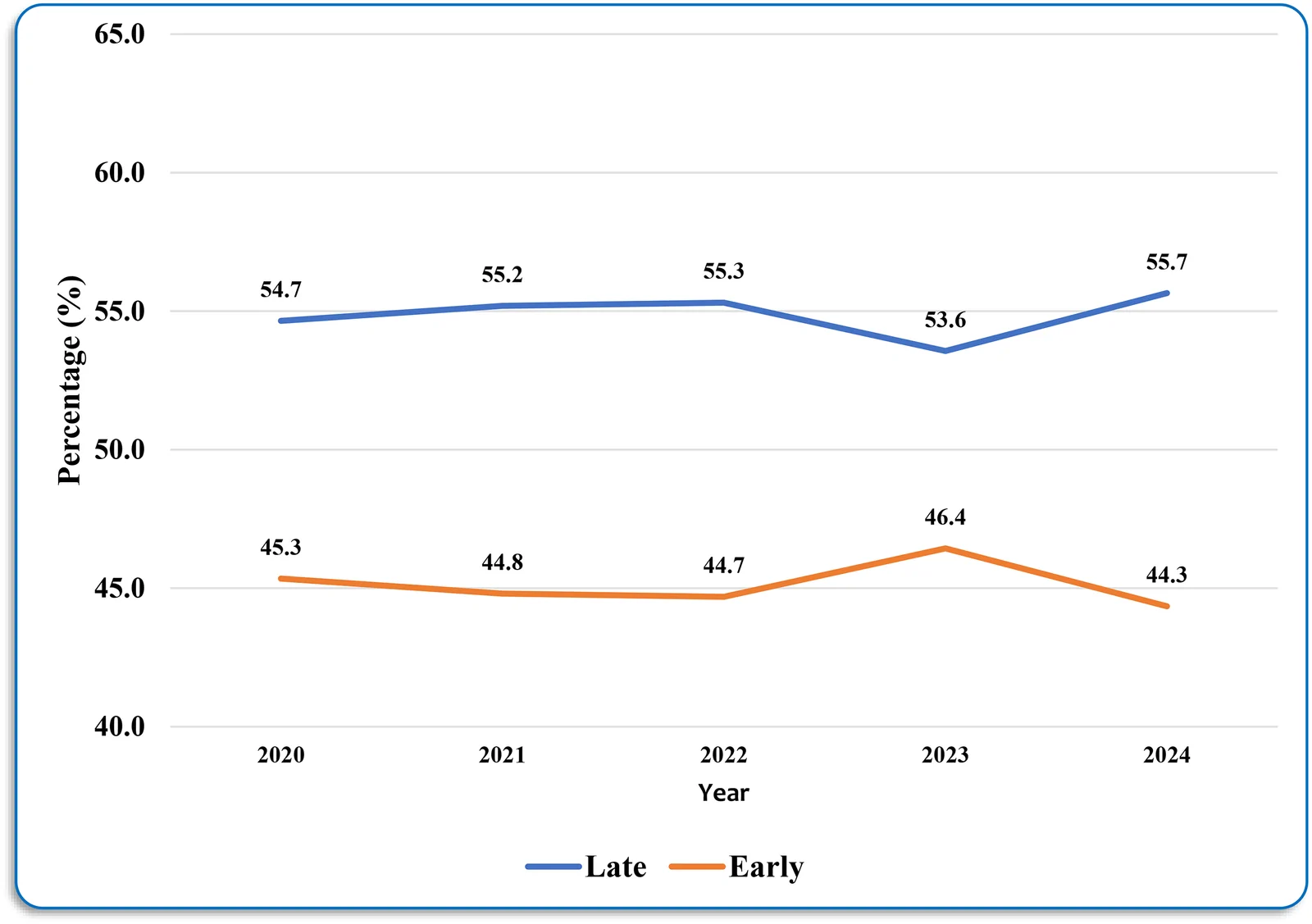
Trends in early antenatal care initiation by year.

### Prevalence of early ANC initiation by type of health facility

Among the three health facilities considered for the study, the highest prevalence of early ANC initiation was observed at Gbi-Wegbe Health Center (48.4%), followed by Volta Regional Hospital (46.4%). The lowest prevalence was found in Adabraka Health Center (39.9%) (Fig 3).

**Fig 3:**
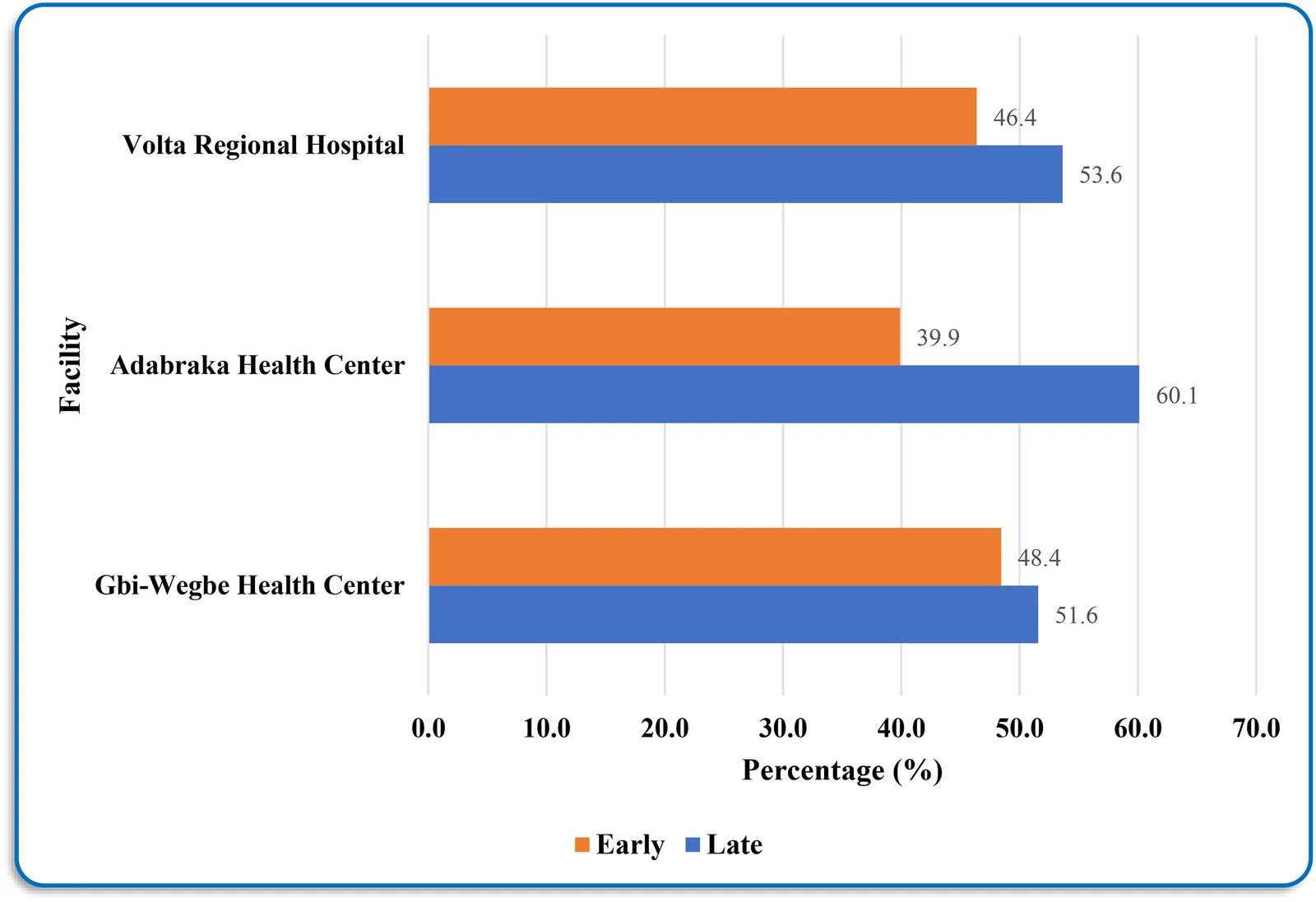
Proportion of early ANC initiation across health facilities.

### Prevalence of early ANC initiation by year and type of health facility

Fig 4 presents the trends in early ANC initiation from 2020 to 2024 across health facilities. Timely ANC initiation varied across facilities and years throughout the study period. At Gbi-Wegbe Health Center, early initiation peaked in 2021 (55.4%) but had already been relatively high in 2020 (51.9%), before declining progressively through 2022 (50.6%), 2023 (45.7%), and 2024 (40.8%), with an overall rate of 48.4%. Adabraka Health Center recorded its lowest early initiation in 2021 (28.5%), following a moderate start in 2020 (40.5%), before recovering gradually to 43.3% by 2024, with an overall rate of 39.9%. Volta Regional Hospital maintained relatively stable rates throughout, ranging from 42.4% in 2022 to a peak of 53.6% in 2023, before declining slightly to 47.1% in 2024, with an overall rate of 46.4%. No facility consistently achieved early initiation above 50% across all five years.

**Fig 4:**
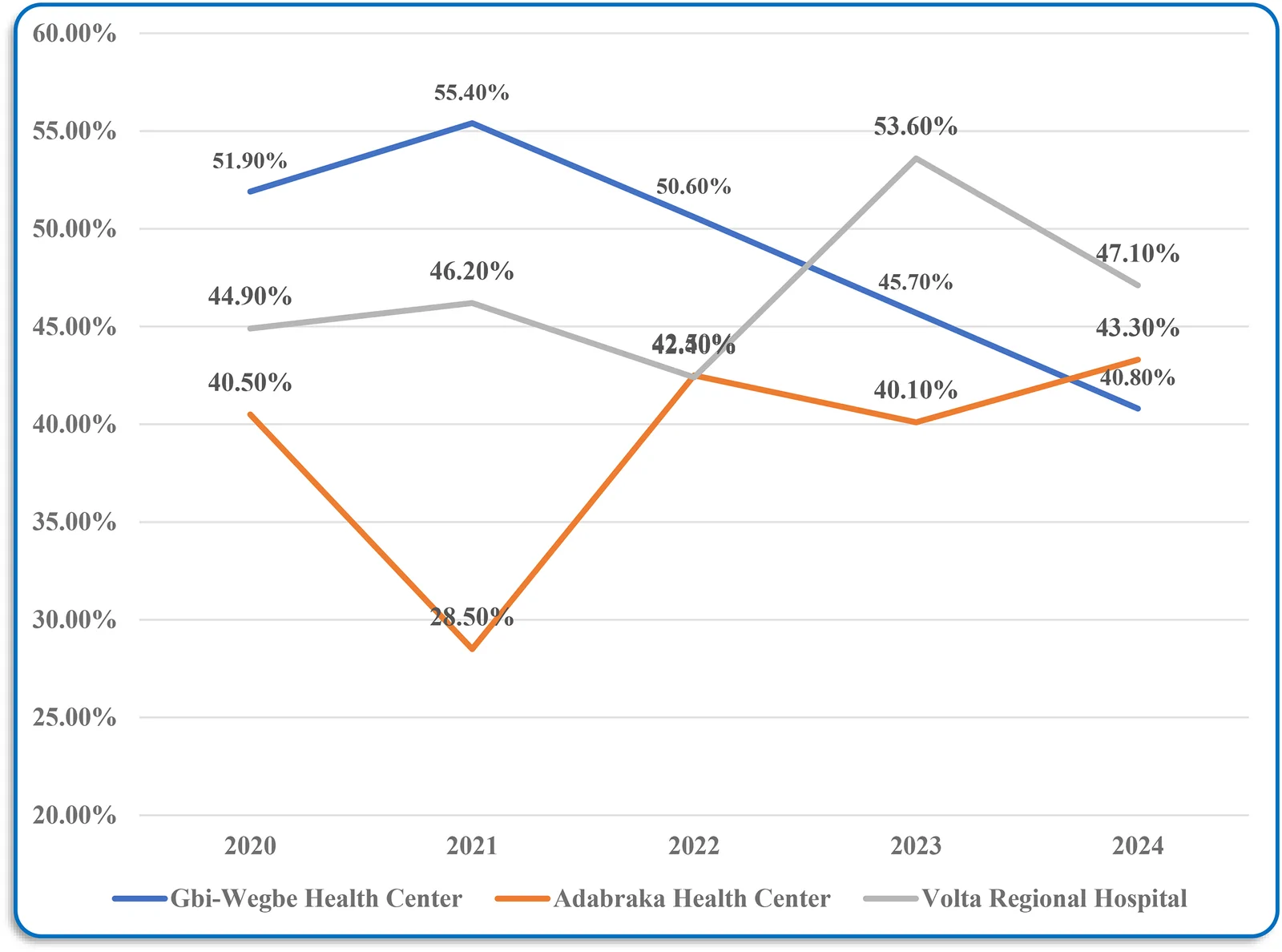
Proportion of early ANC initiation across years and health facilities.

### Comparison of early ANC initiation between the COVID-affected period (2020–2021) and the recovery period (2022–2024)

Table 2 presents the facility-level prevalence estimates and period-specific odds ratios.A pooled logistic regression model incorporating period-by-facility interaction terms revealed significant heterogeneity in the period effect across facilities (LR chi2(13) = 149.92, p < 0.001). At Gbi-Wegbe Health Center, women in the post-COVID-19 period had 27% lower odds of early initiation compared to the COVID-19 period (OR = 0.73; 95% CI: 0.58–0.92; p = 0.008). At Adabraka Health Center, the post-COVID-19 period was associated with more than twice the odds of early initiation relative to the COVID-19 period at Gbi-Wegbe Health Center (OR = 2.14; 95% CI: 1.51–3.03; p < 0.001). At Volta Regional Hospital, the post-COVID-19 period was also associated with significantly higher odds of early initiation (OR = 1.54; 95% CI: 1.17–2.02; p = 0.002).

**Table 2:** Comparison of early ANC initiation between the COVID-affected period (2020–2021) and the recovery period (2022–2024) by health facility.

Facility	2020–2021 early %	2022–2024 early %	Change	OR (95% CI)	p-value	Significance
**Gbi-Wegbe Health Center**	54.3%	46.0%	▼ 8.3pp	0.73 (0.58–0.92)	0.008	Significant
**Adabraka Health Center**	31.5%	42.0%	▲ 10.5pp	2.14 (1.51–3.03)	<0.001	Significant
**Volta Regional Hospital**	45.4%	47.6%	▲ 2.2pp	1.54 (1.17–2.02)	0.002	Significant

*pp = percentage points.*

### Factors associated with early ANC initiation among pregnant women

Table 3 presents the results of the multivariable binary logistic regression analysis. Compared to the 13–30 age group, women aged 31–40 years had higher odds of early ANC initiation (OR = 1.15; 95% CI: 1.01–1.31; p = 0.029). Compared to nulliparous women, multiparous women had 24% lower odds (OR = 0.76; 95% CI: 0.66–0.87; p < 0.001), and grand multiparous women had 66% lower odds (OR = 0.34; 95% CI: 0.25–0.47; p < 0.001) of early ANC initiation. Women at the high end of the acceptable range (OR = 1.44; 95% CI: 1.26–1.65; p < 0.001) and those above acceptable/Grade 1 hypertension (OR = 1.95; 95% CI: 1.34–2.83; p < 0.001) had higher odds of early ANC initiation compared to those in the acceptable range. The facility of ANC attendance also showed significant differences. Compared to Gbi-Wegbe Health Center, women attending Adabraka Health Center (OR = 0.71; 95% CI: 0.62–0.82; p < 0.001) and Volta Regional Hospital (OR = 0.88; 95% CI: 0.78–1.00; p = 0.049) were less likely to initiate ANC early. Multicollinearity among predictor variables was assessed using variance inflation factors (VIFs), with values ranging from 1.00 to 1.73 and a mean VIF of 1.31, indicating low multicollinearity and supporting stable, interpretable estimates. The goodness-of-fit of the final model was assessed using the Hosmer-Lemeshow test, which yielded a chi-square statistic of 5.02 with 8 degrees of freedom (p = 0.756), indicating adequate model fit. The likelihood ratio test confirmed that the full multivariable model fitted the data significantly better than the null model (LR chi2(10) = 128.45, p < 0.001), supporting the overall adequacy of the model specification despite the modest pseudo R² (0.015). The area under the Receiver Operating Characteristic curve (AUC = 0.58) indicated limited discriminatory ability. The classification table showed that the model correctly classified 56.92% of observations, with a specificity of 86.97% and a sensitivity of 20.40%, suggesting the model performs better at identifying late initiators than early initiators.

**Table 3:** Factors associated with early ANC initiation among pregnant women.

Variable	ANC Initiation		Chi-square, p-value	cOR (95% CI), p-value	aOR (95% CI), p-value
	Late (n, %)	Early (n, %)			
**Age**			6.69, 0.035		
14-30	2281 (66.2)	1911 (67.4)		1.00	1.00
31-40	1073 (31.2)	875 (30.9)		0.97 (0.87, 1.08), 0.624	1.15 (1.01, 1.31), 0.029
41-49	91 (2.6)	48 (1.7)		0.63 (0.44, 0.90), 0.011	0.87 (0.59, 1.27), 0.462
**Parity**			53.59, <0.001		
Nullipara	1021 (29.6)	943 (33.3)		1.00	1.00
Primipara	850 (24.7)	781 (27.6)		0.99 (0.87, 1.13), 0.938	0.97 (0.85, 1.11), 0.706
Multipara	1396 (40.5)	1049 (37.0)		0.81 (0.72, 0.92), 0.001	0.76 (0.66, 0.87), <0.001
Grand Multipara	178 (5.2)	61 (2.1)		0.37 (0.27, 0.50), <0.001	0.34 (0.25, 0.47), <0.001
**Hypertension status (mmHg)**			42.17, <0.001		
Acceptable	2868 (83.2)	2183 (77.0)		1.00	1.00
High end of acceptable	516 (15.0)	573 (20.2)		1.46 (1.28, 1.66), <0.001	1.44 (1.26, 1.65), <0.001
Above acceptable/Grade 1	49 (1.4)	71 (2.5)		1.90 (1.32, 2.75), 0.001	1.95 (1.34, 2.83), <0.000
Moderately high/Grade 2 & High Grade 3	12 (0.4)	7 (0.3)		0.77 (0.30, 1.95), 0.577	0.81 (0.31, 2.09), 0.659
**Facility**			26.48, <0.001		
Gbi-Wegbe Health Center	726 (21.1)	682 (24.1)		1.00	1.00
Adabraka Health Center	990 (28.7)	657 (23.2)		0.71 (0.61, 0.82), <0.001	0.71 (0.62, 0.82), <0.001
Volta Regional Hospital	1729 (50.2)	1495 (52.7)		0.92 (0.81, 1.04), 0.195	0.88 (0.78, 1.00), 0.049

*1.00 = Reference category.*

### Facility-stratified subgroup analysis of determinants of early ANC initiation

Table 4 presents the facility-stratified subgroup analysis of determinants of early ANC initiation. Grand multiparity was the only predictor consistent across all three facilities, confirming it as the most robust determinant of late initiation. Maternal age was significant only at Volta Regional Hospital (OR = 1.27; p = 0.006) and absent at the two primary care facilities. The hypertension effect was most pronounced at Adabraka Health Center for Grade 1 hypertension (OR = 3.64; p = 0.007) compared to Volta Regional Hospital (OR = 2.00; p = 0.003) and Gbi-Wegbe Health Center where it was non-significant.

**Table 4:** Facility-stratified subgroup analysis of determinants of early ANC initiation.

Variable	Gbi-Wegbe Health Center (n = 1,408)		Adabraka Health Center (n = 1,647)		Volta Regional Hospital (n = 3,224)	
	OR (95% CI)	p-value	OR (95% CI)	p-value	OR (95% CI)	p-value
**Maternal Age (Ref: 14–30 years)**						
31–40 years	0.97 (0.73–1.30)	0.833	1.05 (0.82–1.36)	0.687	1.27 (1.07–1.51)	0.006
41–49 years	1.06 (0.47–2.36)	0.889	0.49 (0.21–1.12)	0.089	1.01 (0.60–1.70)	0.957
**Parity (Ref: Nullipara)**						
Primipara	1.20 (0.90–1.59)	0.215	1.00 (0.77–1.30)	0.996	0.88 (0.74–1.06)	0.195
Multipara	1.02 (0.76–1.36)	0.919	0.79 (0.60–1.03)	0.078	0.67 (0.55–0.80)	<0.001
Grand Multipara	0.33 (0.16–0.65)	0.002	0.46 (0.25–0.84)	0.013	0.31 (0.19–0.49)	<0.001
**Hypertension status (Ref: Acceptable)**						
High end of acceptable	1.70 (1.27–2.27)	<0.001	1.15 (0.86–1.55)	0.339	1.48 (1.24–1.76)	<0.001
Above acceptable/Grade 1	0.89 (0.35–2.28)	0.807	3.64 (1.42–9.31)	0.007	2.00 (1.27–3.17)	0.003
Moderately high/Grade 2 & High Grade 3	2.25 (0.20–25.23)	0.510	1.08 (0.25–4.64)	0.912	0.39 (0.08–1.95)	0.253
**Model fit statistics**						
LR chi²	32.31	<0.001	24.54	0.002	65.8	<0.001
Pseudo R²	0.017		0.011		0.015	

*Ref = Reference category.*

To formally test whether associations differed across facilities, pooled logistic regression models incorporating facility-by-predictor interaction terms were fitted. The age-by-facility interaction model revealed no statistically significant interactions (all p > 0.05; LR chi2(14) = 131.30, p < 0.001), indicating that the effect of maternal age does not differ significantly across facilities. Similarly, the parity-by-facility interaction model yielded no significant interaction terms (all p > 0.05; LR chi2(16) = 133.64, p < 0.001), formally confirming that the parity gradient operates consistently across all three facilities. The hypertension-by-facility interaction model identified one statistically significant interaction: women with above acceptable/Grade 1 hypertension attending Adabraka Health Center had significantly higher odds of early initiation compared to the reference group (OR = 3.90; 95% CI: 1.04–14.61; p = 0.044), formally confirming that the hypertension effect is significantly more pronounced at this facility. In the fully pooled model incorporating all facility-by-predictor interactions simultaneously, two terms remained statistically significant: multiparous women attending Volta Regional Hospital (OR = 0.66; 95% CI: 0.46–0.93; p = 0.017) and women with Grade 1 hypertension attending Adabraka Health Center (OR = 4.09; 95% CI: 1.08–15.47; p = 0.038), with marginal improvement in model fit (pseudo R² = 0.017 vs 0.015).

### Interaction effect of maternal age and parity on early ANC initiation

To examine whether the effect of maternal age on early ANC initiation differed across parity categories, an interaction term between maternal age and parity was tested and is presented in Table 5. The interaction model showed a marginal improvement in model fit compared to the main effects model (pseudo R^2^ = 0.016 vs 0.015; LR chi2(16) = 140.68, p < 0.001). Two interaction terms were statistically significant. Women aged 31–40 who were multiparous had 39% lower odds of early initiation than expected from their independent effects alone (OR = 0.61; 95% CI: 0.41–0.91; p = 0.016), and those aged 31–40 who were grand multiparous had 71% lower odds (OR = 0.29; 95% CI: 0.11–0.77; p = 0.013), indicating that the generally protective effect of older age on early ANC initiation is substantially attenuated among women with high parity.

**Table 5:** Interaction model: Effect of maternal age × parity interaction on early ANC initiation.

Variable	OR (95% CI)	p-value
**Maternal Age (Ref: 14–30 years)**		
31–40 years	1.66 (1.15–2.39)	0.007
41–49 years	0.46 (0.04–5.23)	0.533
**Parity (Ref: Nullipara)**		
Primipara	0.96 (0.83–1.11)	0.585
Multipara	0.83 (0.72–0.97)	0.021
Grand Multipara	0.73 (0.32–1.68)	0.454
**Age × Parity Interaction Terms**		
31–40 × Primipara	0.83 (0.53–1.27)	0.386
31–40 × Multipara	0.61 (0.41–0.91)	0.016
31–40 × Grand Multipara	0.29 (0.11–0.77)	0.013
41–49 × Primipara	1.24 (0.04–38.30)	0.900
41–49 × Multipara	1.73 (0.15–20.43)	0.662
41–49 × Grand Multipara	1.03 (0.07–14.76)	0.982
**Hypertension Status (Ref: Acceptable)**		
High end of acceptable	1.45 (1.27–1.66)	<0.001
Above acceptable/Grade 1	1.94 (1.33–2.83)	0.001
Moderate-High/Grade 2 & High Grade 3	0.78 (0.30–2.02)	0.611
**Facility of Attendance (Ref: Gbi-Wegbe Health Center)**		
Adabraka Health Center	0.71 (0.61–0.82)	<0.001
Volta Regional Hospital	0.87 (0.77–0.99)	0.031
**Model Fit Statistics**		
LR chi² (df = 16)	140.68	<0.001
Pseudo R²	0.016	—
Number of observations	6,279	—

*Ref = Reference category.*

## Discussion

This study examined the prevalence and trends of early initiation of ANC among pregnant women attending selected health facilities in the Hohoe Municipality from 2020 to 2024. Late initiation of ANC remained more common than early initiation, with 45.1% of women initiating ANC during the first trimester. Temporal patterns varied across the study period and between facilities. Facility-stratified subgroup analysis further revealed that grand multiparity was the only determinant consistent across all three facilities, while the effects of maternal age and hypertension status varied by facility. Factors such as age, parity, hypertension status, and facility of ANC attendance are associated with early ANC initiation. These findings provide valuable insights into ANC utilisation patterns in the Hohoe Municipality and offer a foundation for understanding the structural and behavioural factors influencing maternal health-seeking behaviour.

More than half of the women in this study initiated ANC late, indicating that early engagement with maternal health services remains suboptimal. This finding is consistent with evidence from facility-based studies across sub-Saharan Africa and other LMICs. In western Ethiopia, 56.8% of pregnant women initiated ANC late in a multi-centred cross-sectional study [29]. In Eritrea, 59% of pregnant women booked their first ANC visit after the recommended period in a cross-sectional facility-based study [30]. In southwest Ethiopia, 71.2% of women booked their first ANC visit late, with perceived booking time beyond 12 weeks of gestation and lack of prior ANC attendance as the strongest predictors in a cross-sectional study [31]. In Ethiopia, a multilevel analysis of the 2016 Demographic and Health Survey found a delayed first ANC booking prevalence of 67.3%, with multiparous and grand multiparous women having significantly higher odds of delayed booking compared to nulliparous women [32]. These findings suggest broader structural challenges affecting maternal healthcare access across many sub-Saharan African and LMIC contexts, including limited health system accessibility, sociocultural beliefs about pregnancy care, and delayed recognition of pregnancy.

The prevalence of early ANC initiation in this study was lower than that reported in some Ghana-specific and other LMIC analyses. Using data from the Ghana Maternal Health Survey, 66% of women initiated ANC during the first trimester [13]. In a nationally representative analysis of the Ghana Demographic and Health Survey, 65.1% of women started ANC within the recommended period [12]. In Cameroon, 46% of women aged 15–49 attended ANC early in a multilevel analysis of the 2018 Demographic and Health Survey [33]. In Afghanistan, a timely ANC initiation prevalence of 55.8% was recorded among ever-married women in the Afghanistan Health Survey 2018, a figure higher than the current study’s finding, despite well-documented health system challenges in that context [34]. In Bangladesh, only 22.2% of women in the poorest wealth quintile initiated ANC during the first trimester compared to 61.0% among the richest, demonstrating a marked wealth gradient in timely initiation in an analysis of the 2017–18 Demographic and Health Survey [35]. These differences may be attributable to methodological and contextual variations. Nationally representative survey datasets capture broader and more diverse populations, including women from regions with stronger maternal health outreach programmes, whereas facility-level studies reflect local contextual realities such as service accessibility, community awareness, and facility-specific barriers that influence care-seeking behaviour.

Late ANC initiation remained consistently high over the five-year study period, with only a slight decline observed in 2023 and minimal overall improvement across the study period. This suggests that maternal health interventions during the study period may not have significantly changed care-seeking behaviour in the municipality. Gradual improvements in ANC uptake over fifteen years were observed in an analysis of Demographic and Health Survey data from Rwanda, though the pace of change remained slow, highlighting the challenge of altering maternal health behaviour through short-term interventions alone [36]. In a multi-country population-based cross-sectional analysis of Demographic and Health Survey data from the Democratic Republic of Congo, Kenya, and Tanzania between 2007 and 2016, late ANC initiation declined in Tanzania (60.9% to 49.8%) and Kenya (67.8% to 60.5%) but increased in the DRC (56.8% to 61.0%), highlighting that improvements are not uniform even within the same sub-region [37].

The facility-level differences observed in this study further emphasise the significance of local health system dynamics, with late initiation most prevalent at Adabraka Health Center, followed by Volta Regional Hospital and Gbi-Wegbe Health Center. Both individual and facility-level factors were found to influence early ANC initiation in a multilevel analysis in Ethiopia, with women attending certain types of facilities significantly more likely to start ANC early [38]. In a multicentre cross-sectional study in Ghana, negative experiences during ANC visits, including feeling poorly received and spending more than six hours at facilities, substantially increased the likelihood of late ANC initiation [20]. In the Amazon region of Peru, women in urban environments had higher odds of first-trimester ANC attendance, suggesting that facility access shapes initiation patterns in a retrospective matched case-control study [39]. These findings suggest that organisational factors such as waiting times, staff attitudes, and perceived quality of care may determine when women choose to seek antenatal services.

Women aged 31–40 years had significantly higher odds of early ANC initiation compared to those aged 13–30 years in the current study. Greater awareness of pregnancy-related risks, higher decision-making autonomy, and more prior experience with health services among older women may motivate earlier engagement with antenatal care. This finding is consistent with evidence from multiple LMIC settings. In Afghanistan, women aged 30–39 years had higher odds of timely ANC initiation in an analysis of the Afghanistan Health Survey 2018, a finding consistent with the current study despite the very different healthcare context [34]. In rural and peri-urban communities in South Africa, women aged under 20 years had significantly higher odds of late ANC presentation, with adolescents facing compounding barriers, including unplanned pregnancy and limited access, in a cross-sectional study [40]. In Cameroon, women aged 35–39 years had significantly higher odds of early ANC attendance in a multilevel analysis of the 2018 Demographic and Health Survey, further affirming the cross-setting pattern of older age facilitating earlier initiation [33]. In Ghana, older maternal age was associated with higher odds of early ANC initiation, attributed to improved health knowledge and reduced dependence on others for healthcare decisions [12]. The non-significant finding among women aged 41–49 years in the current study may reflect the relatively small number of pregnancies in this age group, limiting statistical power to detect a difference.

A dose-response parity gradient was observed in this study, with primiparous, multiparous, and grand multiparous women showing progressively lower odds of early ANC initiation compared with nulliparous women, suggesting that women with previous childbirth experience may feel less urgency to seek antenatal care early in subsequent pregnancies. In Ghana, women with three or more children had significantly lower odds of initiating ANC early [12], and women with three or four children were more likely to start ANC later than those with one child [20]. In a multilevel analysis of the 2016 Ethiopian Demographic and Health Survey, multiparous women (aOR = 1.21; 95% CI: 1.01–1.45) and grand multiparous women (aOR = 1.50; 95% CI: 1.16–1.93) had significantly higher odds of delayed first ANC booking compared to nulliparous women [32]. In southwest Ethiopia, primigravida women were significantly more likely to initiate ANC timely (aOR = 2.6; 95% CI: 1.4–4.7) in a cross-sectional facility-based study, underscoring that first pregnancy is a stronger motivator for early care-seeking than subsequent pregnancies [41]. In Uganda, 73% of high-parity women delayed seeking their first ANC visit in an analysis of the 2016 Uganda Demographic and Health Survey, confirming the consistent relationship between high parity and late initiation across sub-Saharan Africa [42]. In Afghanistan, women who had given birth at least twice had lower odds of timely initiation in the Afghanistan Health Survey 2018 [34]. In Nigeria, parity-related patterns in ANC timing were also identified, with maternal education, media exposure, and region as uniform predictors across different trimesters of initiation in a secondary analysis of the 2013 Nigeria Demographic and Health Survey [43]. These similarities reflect behavioural patterns in which experienced mothers rely on prior pregnancy knowledge and delay seeking professional care until later stages of pregnancy.

Women with high-normal blood pressure or Grade 1 hypertension were more likely to start ANC early compared to women with acceptable blood pressure in this study, suggesting that perceived health risk may motivate earlier care-seeking. In the Mutaba’ah cohort study in the United Arab Emirates, women with previous miscarriages or infertility treatment were significantly more likely to initiate ANC within the recommended timeframe, supporting the idea that perceived health risks motivate early care-seeking behaviour [44]. In the Amazon region of Peru, women with a history of miscarriage had significantly higher odds of first-trimester ANC attendance in a retrospective matched case-control study, reinforcing the pathway from perceived clinical risk to earlier engagement with antenatal services [39].

Women attending Adabraka Health Center and Volta Regional Hospital had lower odds of early ANC initiation compared with those attending Gbi-Wegbe Health Center, reflecting differences in accessibility, perceived service quality, or organisational factors such as waiting time and patient load. Facility-level characteristics significantly shaped ANC initiation patterns in a multilevel analysis in Ethiopia, and long waiting times during ANC visits strongly predicted late initiation in a multicentre cross-sectional study in Ghana [20,38]. In a cross-sectional study in Jimma Zone public hospitals in southwest Ethiopia, a travel time greater than 60 minutes to the health facility was strongly associated with late ANC initiation, underscoring how facility accessibility shapes initiation timing beyond individual-level characteristics [45]. These findings suggest that improving the patient experience at health facilities may play an important role in encouraging women to seek care earlier in pregnancy.

Heterogeneous patterns in early ANC initiation emerged across the COVID-19 (2020–2021) and post-COVID-19 (2022–2024) periods, despite no statistically significant change in overall initiation rates between the two periods. Early initiation declined significantly at Gbi-Wegbe Health Center (54.3% to 46.0%), improved significantly at Adabraka Health Center (31.5% to 42.0%), and increased significantly at Volta Regional Hospital (45.4% to 47.6%). The influence of the COVID-19 pandemic on primary maternal and child health services has been mixed across sub-Saharan Africa, with attendance declining at some facilities while others experienced no significant change or even improvement in a scoping review [46]. Fear of contracting the virus, limited access to transport, lockdown policies, and weaknesses in the health system were identified as key barriers to accessing maternity care during COVID-19 in sub-Saharan Africa, likely contributing to the decline observed at Gbi-Wegbe Health Center [47]. In Kenya, women who delivered during the COVID-19 period had significantly higher odds of delayed ANC initiation, with nearly half reporting that the pandemic affected their ability to access ANC in a cross-sectional study, a pattern that mirrors the disruption observed at Gbi-Wegbe Health Center [48]. The subsequent significant improvements at Adabraka Health Center and Volta Regional Hospital in the post-COVID-19 period suggest a recovery effect, with strengthening community involvement in maternal health service delivery recommended as a strategy to improve resilience and uptake following public health emergencies [47].

Grand multiparity was the only predictor consistent across all three facilities in the facility-stratified subgroup analysis, confirming it as the most robust determinant of late initiation regardless of facility type or level of care. In Uganda, 73% of high-parity women delayed their first ANC visit across diverse geographic and facility contexts in an analysis of the 2016 Uganda Demographic and Health Survey, further supporting the cross-facility universality of this finding [42]. Maternal age was a significant predictor only at Volta Regional Hospital and absent at the two primary care facilities, suggesting that the age effect on early initiation may be more pronounced in referral settings where women with more complex clinical profiles and greater health awareness are concentrated. The hypertension effect was most pronounced at Adabraka Health Center for Grade 1 hypertension compared to Volta Regional Hospital and was non-significant at Gbi-Wegbe Health Center, suggesting that women with elevated blood pressure attending Adabraka Health Center may represent a particularly high-risk subgroup motivated by perceived clinical need to seek early care. Formal pooled logistic regression models incorporating facility-by-predictor interaction terms confirmed that the age and parity effects did not differ significantly across facilities, while the hypertension effect at Adabraka Health Center was formally confirmed as significantly stronger than at Gbi-Wegbe Health Center, providing statistical evidence that the observed facility-level heterogeneity in hypertension effects represents a genuine interaction rather than a descriptive artefact. These facility-level differences highlight that determinants of early ANC initiation are not uniform across settings and point to the need for facility-specific strategies alongside population-level interventions.

The persistence of late ANC initiation in this study has important implications for maternal health policy and programme planning. Early ANC initiation plays a crucial role in detecting pregnancy-related complications, delivering preventive interventions, and promoting maternal and foetal well-being. Delayed entry into antenatal care may limit opportunities for early screening for hypertension, infections, and other pregnancy complications. Consequently, efforts to improve early ANC uptake should focus not only on individual behaviour but also on structural barriers within health systems. Strengthening community education programmes may help improve awareness about the benefits of early ANC, and community health workers and maternal health campaigns can play an important role in promoting early recognition of pregnancy and encouraging early care-seeking. At the health system level, interventions to reduce waiting times, improve patient-provider communication, and strengthen service quality may also enhance women’s willingness to initiate ANC early.

These efforts align with the broader objectives of the global development agenda, particularly the United Nations’ SDG 3, which seeks to ensure healthy lives and promote well-being for all. Improving early access to antenatal care directly contributes to reducing maternal and neonatal morbidity and mortality, key targets within the global maternal health strategy. By addressing the barriers that delay ANC initiation, health systems can move closer to achieving universal access to essential maternal health services.

## Strength and limitations

The multi-facility repeated record review cross-sectional design enabled the inclusion of diverse healthcare settings within Hohoe Municipality, capturing ANC delivery across rural, peri-urban, and referral-level contexts. By purposively selecting Gbi-Wegbe Health Center, Adabraka Health Center, and Volta Regional Hospital, the study accounted for both routine and complex maternal cases, reflecting real-world ANC utilisation patterns. A major strength is the census approach, which included all eligible ANC records from January 2020 to December 2024. This maximised statistical power, ensured representativeness, and enabled the identification of temporal trends in early and late ANC initiation. The study also implemented rigorous data quality measures, including standardised extraction sheets, trained research assistants, and principal investigator cross-checks. Alignment with STROBE guidelines further enhanced validity and reliability. The statistical analysis was strengthened by careful assessment of multicollinearity among explanatory variables using variance inflation factors (VIFs). Low VIF values (mean = 1.31) confirmed minimal intercorrelation, supporting stable and interpretable regression estimates for determinants of early ANC initiation.

Nevertheless, limitations exist. The study relies exclusively on variables available in routine ANC registers. Key established predictors of early ANC initiation, including educational level, marital status, socioeconomic status, distance to facility, employment status, and cultural or religious beliefs, were absent from the analysis. These are among the variables most consistently associated with ANC timing in the broader literature, and their absence limits the explanatory scope of the regression model and likely contributes to its modest discriminatory ability. Purposively selecting three facilities from twenty-one in the municipality means the majority of primary care facilities are not represented. While the selected facilities provide a spectrum of care levels, findings cannot be considered representative of ANC patterns across the entire municipality, and generalisability is appropriately bounded to the three study facilities and similar settings. As a retrospective record-review study, gestational age at the first ANC visit was estimated from clinical records in facility registers, most likely derived from the last menstrual period rather than confirmed by first-trimester ultrasound. In resource-limited settings, last menstrual period recall is subject to recall bias and irregular cycle variation, introducing the possibility of misclassification of the primary outcome: some women classified as late initiators may have initiated early, and vice versa. Although the Hosmer-Lemeshow test indicated adequate model fit, the modest pseudo R^2^ (0.015) and limited discriminatory ability reflected in the area under the Receiver Operating Characteristic curve (AUC = 0.58) suggest that the model explains only a small proportion of the variation in early ANC initiation, reflecting that important predictors such as educational level, socioeconomic status, and cultural beliefs were not captured in the available facility records. Finally, while the repeated cross-sectional design enables the examination of temporal trends across the study period, it remains observational in nature and precludes causal inference; observed associations cannot establish directionality.

## Conclusion

This study reveals that more than half of pregnant women in Hohoe Municipality initiate ANC late. Facility-stratified analysis across the COVID-19 and post-COVID-19 periods revealed heterogeneous patterns, with early initiation declining significantly at Gbi-Wegbe Health Center and increasing significantly at both Adabraka Health Center and Volta Regional Hospital, underscoring the differential impact of the pandemic on ANC timing across facility types. Key determinants identified include age, parity, hypertension, and the facility attended, highlighting the complex interplay of individual, obstetric, and service-level influences. These findings are consistent with broader regional studies, emphasising the ongoing challenge of ensuring early maternal care. To address this, the Ghana Health Service (GHS), the Ministry of Health, and Municipal Health Directorates should strengthen community outreach programmes to target multiparous and high-risk women with tailored health education and to raise awareness among women of reproductive age of the importance of early pregnancy screening, particularly for hypertensive disorders. Given the heterogeneity of determinants across facilities, interventions should be tailored to each facility’s specific context rather than relying solely on uniform, municipality-wide strategies. Efforts should focus on improving facility accessibility, reducing waiting times, providing mobile ANC clinics, and training staff to deliver patient-centred, culturally sensitive care. Future research should examine sociocultural norms, financial barriers, and health system practices that influence ANC timing, using longitudinal designs to evaluate the impact of targeted interventions. Understanding these factors will inform context-specific strategies to improve early ANC uptake and advance maternal and neonatal health outcomes, both within the Hohoe Municipality and across similar resource-limited settings in sub-Saharan Africa.

## Supporting information

S1 file STROBE Checklist. (DOCX)

## Abbreviations

ANC: Antenatal Care
AOR: Adjusted Odds Ratio
AUC: Area Under Curve
COR: Crude Odds Ratio
GHS: Ghana Health Service
MoH: Ministry of Health
ROC: Receiver Operating Characteristic
SDGs: Sustainable Development Goals
STROBE: Strengthening the Reporting of Observational Studies in Epidemiology
UNFPA: United Nations Population Fund
UNICEF: United Nations Children’s Fund
VIF: Variance Inflation Factors
WHO: World Health Organization
