# Supplementary material for: Early antenatal care initiation and associated factors in Hohoe Municipality, Ghana: A five-year multi-facility repeated cross-sectional record review study
Source: PLoS One. 2026 Nov 4;21(11):e0356427. doi: 10.1371/journal.pone.0356427 (PMC13734592; doi:10.1371/journal.pone.0356427)
Supplement: Supplementary file 2 [file pone.0356427.s002.docx]

**RESPONSE TO REVIEWERS’ COMMENTS**

**Reviewer #1:**

There are some suggestions to make your study stronger.

There is a incosistency between abstract and results section.

Response: Inconsistencies have been revised accordingly.

The introduction is too long, you can make it concise.

Response: Background has been reduced to be more concise.

The discussion section is very good but it is better to discuss about your unexpected results (for example: hypertension and early ANC).

The implication of the study is too general, make it specific.

Conclusion is too general and you have to write based on your findings.

Response: Conclusion has been revised accordingly.

**Reviewer #2: General assessment**

Strengths

Relevant maternal/public health topic in sub-Saharan Africa.

Large retrospective dataset over 5 years.

Multi-facility design including primary and referral settings.

Use of adjusted logistic regression.

Trend component adds some value.

Major concerns

Novelty is limited

Similar studies already exist nationally in Ghana and regionally.

Authors must better justify why Hohoe municipality data adds substantial new evidence.

Question to authors:

What specific knowledge gap does this study fill beyond prior Ghana DHS analyses and nearby district studies?

Response: The background has been reduced to be more concise, and arguments have been put together to strengthen the work.

TITLE AND ABSTRACT

Title (Lines 1–2)

Comment: Title is clear but somewhat descriptive and lengthy.

Suggested revision:

Early antenatal care initiation and associated factors in Hohoe Municipality, Ghana: a five-year retrospective facility-based study

Response: The study title has been revised accordingly

Abstract

Lines 24–28 (Background)

Comment: Good rationale.

Question:

Why were only selected facilities included instead of all 21 facilities in Hohoe Municipality?

This may affect representativeness.

Response: The three facilities were purposively selected to represent the different levels of healthcare delivery within Hohoe Municipality, ranging from primary care in rural and peri-urban communities to referral-level hospital care. Together, they represent the major providers of antenatal care services in the municipality. Including all 21 facilities would have introduced considerable variability in record-keeping quality and risked compromising data accuracy. The limitations section has been revised to explicitly acknowledge that findings cannot be considered representative of ANC patterns across the entire municipality, and that generalisability is appropriately bounded to the three study facilities and similar settings.

Lines 29–35 (Methods)

Comment: Methods are generally clear.

Concern: Authors mention retrospective analysis but not missing data handling.

Question:

How many records were excluded due to incompleteness or duplication before arriving at 6,279?

A flow diagram would improve transparency.

Response: A flow diagram has been provided accordingly.

Lines 37–39 (Results)

Major issue: inconsistency

Abstract says:

2020 = 44.3%

2024 = 46.4%

But Results section Figure 3 states:

2020 = 45.3%

2024 = 44.3%

These are inconsistent.

Response: Years and percentages have been correctly effected.

Question to authors:

Please reconcile discrepancies in yearly prevalence values between abstract and Results/Figure 3.

This is a serious reporting issue.

Response: Years and percentages have been correctly effected.

Lines 40–51

Comment: Too many adjusted ORs in abstract.

Recommendation:

Reduce detailed estimates and retain only strongest predictors.

Response: Estimates have been reduced with a focus on only the strongest predictors.

Lines 53–58 (Conclusion)

Concern: “Reducing high parity” is not an immediate intervention target.

Parity is a reproductive history variable, not easily modifiable.

Suggested wording:

“…targeted interventions focusing on reproductive counseling, early pregnancy recognition, and anemia prevention…”

Response: The conclusion has been revised to reflect the suggestion accordingly.

INTRODUCTION / BACKGROUND

Lines 62–83

Comment: Background is comprehensive but verbose.

Could be shortened by ~20%.

Examples:

maternal mortality discussion is overly long for a local facility study.

Lines 79–80

Issue: suspicious estimate

Authors report:

“0.2% early initiation in Niger to 16.6% in Liberia”

These seem implausibly low.

Question:

Please verify these estimates and citations.

Potential citation or interpretation error.

Response: After reducing the background by a percentage, things have been made more precise, and the estimate has been taken off.

Lines 99–113

Comment: This is where novelty should be strengthened.

Currently:

“Hohoe lacks retrospective analysis”

This alone is weak.

Authors should explicitly argue:

post-COVID period,

facility-level trends,

clinical predictors unavailable in DHS.

Response: The background has been reduced to be more precise, and arguments have been put together to strengthen the work.

METHODS

Study design (Lines 116–125)

Line 117

Comment: “retrospective cross-sectional” acceptable.

But because 5-year records analyzed:

Could also be described as repeated cross-sectional record review.

Response: Study design and all other areas within the manuscript have had changes effected accordingly.

Line 124

Question:

Please provide STROBE checklist as supplementary file.

Response: The STROBE checklist has been provided accordingly as a supplementary file.

Study site (126–139)

Too detailed.

Geographical boundaries can be condensed.

No need for long municipal description.

Response: The study site detail has been condensed and made more precise.

Inclusion/exclusion (147–163)

Major missing information

Authors say incomplete records excluded.

Question:

How many records were excluded for:

missing gestational age,

transfer-in cases,

duplicates?

Need explicit numbers.

Response: The exclusion criteria described in the methods were applied prospectively during data extraction rather than post-hoc. Transfer-in cases were not captured, duplicates were prevented through built-in validation, and only complete records within the study period were extracted. All 6,279 retrieved records were therefore included in the final analysis, and the methods have been revised to reflect this accurately.

Sample size (164–170)

Fine.

But authors should clarify denominator:

Were all first ANC attendees included only?

Response: The denominator has been explicitly clarified in the revised manuscript. The sample comprises all women attending their first ANC visit at the selected facilities during the study period. Repeat visits and subsequent ANC contacts by the same woman were not included in the analysis.

Study variables (171–195)

Major methodological concern

Lines 179–184

Authors include:

parity

gravidity

Both are highly correlated conceptually.

Even though VIF is low, this may introduce conceptual redundancy.

Question:

Why were parity and gravidity included simultaneously in the same regression model?

This may complicate interpretation.

Response: Gravidity has been excluded from the bivariate and final multivariable model.

Line 183–184

Problem:

“We coded gravida into 0 = primigravida and 1 = multigravida.”

This is not ideal wording.

Use:

primigravida vs multigravida.

Response: Primigravida vs multigravida has been used accordingly.

Lines 187–193

Blood pressure categories included.

But causal interpretation is problematic.

BP measured at first visit is concurrent with outcome timing.

Thus hypertension cannot truly predict early ANC.

It is measured after care-seeking.

Major concern

Question:

How do authors justify interpreting blood pressure as a determinant rather than a concurrent clinical characteristic?

Response: Blood pressure was available in the antenatal care records retrieved. The blood pressure data measured the hypertension status of the women prior to pregnancy, and not pregnancy-induced hypertension. This made it eligible for inclusion in the country.

Same issue applies to hemoglobin.

Response: Haemoglobin has been excluded from the bivariate and final multivariable model.

Sampling/data collection (196–219)

Well described.

But “representative” claim is too strong since facilities purposively selected.

Suggested revision:

Replace “representative” with “captures diverse care settings.”

Response: Replacement has been effected accordingly

Validity (220–232)

Line 230

“maximum agreement”

Not scientific wording.

Need kappa or percent agreement.

Question:

What was inter-rater reliability percentage?

Response: The phrase “maximum agreement” has been replaced with a precise description of the data quality process employed. A source document verification procedure was used, whereby a second researcher cross-checked all entered data against original ANC registers before final submission.

DATA ANALYSIS

Lines 233–248

Major issue

Bivariate selection based on significance only.

This is not ideal.

Variables should be selected based on theory/literature.

Question:

Were all clinically relevant covariates included regardless of bivariate significance?

Response: The methods have been updated to clarify that all clinically relevant covariates were retained in the multivariable model regardless of bivariate significance.

Missing model diagnostics

Need:

goodness of fit (Hosmer-Lemeshow or equivalent)

ROC/AUC maybe optional.

Response: The Hosmer-Lemeshow goodness-of-fit test and ROC/AUC have been conducted and reported.

RESULTS

Figure 2

Typo:

“Late Eearly”

Needs correction.

Response: Correction has been effected accordingly.

Figure 3

Trend labels inconsistent with abstract.

Must correct.

Response: Correction has been effected accordingly.

Figure 5

Major concern

Facility-specific yearly proportions appear extreme:

2020:

Gbi-Wegbe = 11%

Adabraka = 5.5%

Volta Regional = 83.5%

This is highly unusual.

Could indicate denominator/coding issue.

Question: Please explain why Volta Regional Hospital had 83.5% early initiation in 2020 while other facilities were <11%.

Potential data anomaly.

Response: Thank you for the observation. However, it is not a data anomaly. At the apex of the referral hierarchy, Volta Regional Hospital functions as the main secondary referral facility, which is more resourced in operation and function compared to the health centres.

Table 2

Major interpretation issue

Authors state: multigravida more likely early (aOR 1.67) higher parity less likely early

This seems contradictory. Because gravidity and parity are related.

Likely model instability due to collinearity/conceptual overlap.

Question: Did authors test model excluding either parity or gravidity?

Highly recommended.

Response: Per the recommendation, Gravidity has been excluded from the bivariate and final multivariable models, and Parity has been retained.

DISCUSSION

Lines 375–381

Interpretation of multigravida contradicts parity discussion.

Needs reconciliation.

Authors currently say: prior experience delays care AND prior experience improves early care.

Both cannot be simultaneously generalized without nuance.

Need clearer explanation.

Response: Gravidity has been excluded from the bivariate and final multivariable model; it only remains descriptive.

Lines 383–391

Problematic causal language.

Authors imply anemia influences ANC timing.

Actually reverse may be possible: late ANC may increase probability of anemia detection later.

Recommendation: Reframe as association only.

Response: Haemoglobin status has been excluded from the final multivariable model

CONCLUSION

Lines 449–461

Issue: Recommendations exceed study evidence.

Example: mobile ANC clinics not directly studied.

Recommendation should align with findings.

Suggested: targeted maternal education, improved facility experience, screening awareness.

Response: Suggestions have been fully integrated into the manuscript.

ABBREVIATIONS

Line 465

Error: MoH incorrectly expanded as:

Ministry of Finance

Should be: Ministry of Health

Response: The error has been corrected accordingly

REFERENCES

Need formatting consistency check: capitalization inconsistent, journal styles inconsistent.

Response: All references have been corrected accordingly.

**Reviewer #3:** This manuscript reports a retrospective, multi-facility, cross-sectional study examining the prevalence, annual trends, and determinants of early antenatal care (ANC) initiation among 6,279 pregnant women attending three public health facilities in Hohoe Municipality, Ghana, between January 2020 and December 2024.

Comment 1

The abstract states that early ANC initiation “slightly increased from 44.3% in 2020 to 46.4% in 2024”. However, Figure 3 and the accompanying results text show the opposite direction: 2020 started at 45.3%, dipped to 44.7%-44.8% in 2021-2022, rose to a peak of 46.4% in 2023, and then fell back to 44.3% in 2024. The 2024 figure of 44.3% is therefore lower than the 2020 figure of 45.3%, and the peak year was 2023, not 2024.

Response: Years and percentages have been correctly effected.

Comment 2

Figure 5 reports that Volta Regional Hospital had an early ANC initiation rate of 83.5% in 2020, which then declined sharply to 44.7% by 2024. At the same time, Figure 4 reports the five-year pooled early initiation rate at Volta Regional Hospital as 46.4%, and Gbi-Wegbe Health Center as 48.4%. If Volta Regional Hospital began at 83.5% in 2020, the pooled five-year figure of 46.4% implies very low rates in subsequent years to average down to that level. Yet Gbi-Wegbe's pooled rate (48.4%) exceeds Volta's (46.4%), despite the latter starting at 83.5%. The manuscript must reconcile these figures explicitly, including the number of records contributed by each facility per year, and provide a narrative explanation for the extreme decline at Volta Regional Hospital.

Response: Thank you for prompting a thorough verification of the facility-level trend data. Upon cross-checking all figures against the raw dataset, the year-by-year narrative figures contained errors and have been completely rewritten to accurately reflect outputs for all three facilities across the five-year period.

Comment 3

The manuscript includes both gravidity (primigravida vs. multigravida) and parity (nullipara, primipara, multipara, grand multipara) as independent predictors in the same multivariable model. These two variables are conceptually and empirically highly correlated: a primigravida woman is by definition nulliparous, and multigravida women are almost always parous. Despite the authors' VIF assessment showing values below 2.42, VIF is not sensitive to perfect or near-perfect category-level collinearity when variables are categorical. The crude odds ratio for gravidity is 1.04 (not significant, p=0.482), yet the adjusted OR is 1.67 (p<0.001), which represents a large and statistically significant shift that is unusual and may reflect collinearity-driven instability rather than a genuine independent effect. The authors must provide a conceptual and statistical justification for including both variables simultaneously.

Response: In response, gravidity has been removed from the final multivariable model, and Parity has been retained. The model, results, and all relevant sections of the manuscript have been updated accordingly.

Comment 4

The abstract reports the adjusted OR for moderate anaemia as aOR=0.25 (95% CI: 0.22–0.39). However, Table 2 reports this as aOR=0.25 (95% CI: 0.22–0.29). These two confidence intervals have the same lower bound (0.22) but different upper bounds (0.39 in the abstract vs. 0.29 in the table). The correct value must be identified from the Stata output and the discrepancy corrected.

Response: Correction has been effected accordingly to reflect aOR=0.25 (95% CI: 0.22–0.29), instead of aOR=0.25 (95% CI: 0.22–0.39)

The following issues should be corrected to improve accuracy and clarity.

1. The figure legend for Figure 2 (the pie chart) labels the early ANC slice as “Eearly” — this is a typographical error and should read “Early”.

Response: We thank you for this observation. Correction has been effected accordingly.

2. The list of abbreviations defines MoH as “Ministry of Finance” rather than “Ministry of Health”. This should be corrected.

Response: Correction has been effected accordingly.

3. The manuscript describes the data availability as follows: “The datasets used or analysed during the current study are available from the corresponding author on reasonable request.” PLOS ONE requires that underlying data be fully publicly available without restriction, not merely available on request. The authors should either deposit the dataset in a public repository and provide the DOI, or provide a specific justification for why this is not feasible (for example, patient confidentiality concerns under Ghanaian data protection law), as PLOS ONE permits exceptions in documented circumstances.

4. The introduction contains the statement that “43% of pregnant women begin their first antenatal care visit late” globally, citing Reference 5 (Girma et al., 2023, an Addis Ababa facility-based study). A single-city facility study is not an appropriate source for a global statistic. A global or systematic review source should be substituted for this specific claim.

Response: The figures have been verified against the primary source and corrected, with the primary citation as well.

5. The conclusion calls for efforts to focus on “reducing high parity” as an intervention. Parity is not an intervenable exposure in the short term; the intended recommendation appears to be family planning promotion or birth spacing. The conclusion should be revised to reflect a realistic and ethically appropriate intervention target.

Response: The conclusion has been revised to reflect a realistic and ethically appropriate intervention target.

6. Reference 3 is listed as a UNFPA Turkey webpage, cited to support the statistic that 70% of maternal deaths occur in sub-Saharan Africa. A peer-reviewed source or the primary WHO/UNICEF/UNFPA report should be used instead of a country office webpage.

Response: A primary WHO, UNICEF, UNFPA report has been used instead of a country office webpage.

7. The discussion notes that the Volta Regional Hospital decline from 83.5% (2020) to 44.7% (2024) is presented without any substantive discussion of possible causes, despite being the most striking finding in the trend data. Given that this facility contributes over half of the total study sample (3,224 of 6,279 records), its trajectory dominates the pooled figures. A dedicated discussion of plausible explanations is essential for readers to interpret the overall trend correctly.

Response: Upon verification of the facility-level figures against the raw dataset, the previously reported figures of 83.5% in 2020 and 44.7% in 2024 for Volta Regional Hospital were found to be erroneous. The correct figures show a relatively stable trajectory ranging from 45.0% in 2020 to 47.1% in 2024, with no statistically significant period difference.

8. The study period includes the peak COVID-19 years of 2020 and 2021. While the background section discusses COVID-19's impact on ANC in Ghana, the results section makes no attempt to examine whether 2020-2021 patterns differ from 2022-2024, nor is this addressed in the discussion as a limitation on trend interpretation. A brief explicit comment on this is warranted.

Response: A formal period comparison stratified by facility has been conducted and incorporated into the Results and Discussion. While no significant overall difference was found between 2020–2021 and 2022–2024, facility-level analysis revealed significant divergence and a decline at Gbi-Wegbe (p=0.004) and a significant improvement at Adabraka (p=0.001), findings that enrich the interpretation of temporal trends in the manuscript.

The limitations section is present but should be expanded to acknowledge the following:

• The study relies exclusively on variables available in routine ANC registers. Key established predictors of early ANC initiation including education level, marital status, socioeconomic status, distance to facility, employment, and cultural or religious beliefs are entirely absent from the analysis. This is acknowledged briefly but should be foregrounded more prominently, as the regression model explains only part of the variation in early initiation, and the missing variables are among those most consistently associated with ANC timing in the broader literature.

• The purposive selection of three facilities out of twenty-one in the municipality means that the majority of primary care facilities are not represented. The authors argue that the selected facilities provide a spectrum of care, which is reasonable, but the claim that findings are “representative of ANC patterns across the municipality” is overstated given that 18 facilities are excluded. The limitations section should clarify the boundaries of generalisability more precisely.

• The retrospective record-review design means that gestational age at first visit was recorded as a clinical estimate, not confirmed by first-trimester ultrasound. In many resource-limited settings, gestational age is estimated from the last menstrual period, which is subject to recall bias and irregular cycle variation. The potential for misclassification of early versus late initiation, the primary outcome, should be acknowledged.

Response: The limitations section has been substantially revised to address all three points raised, with greater prominence given to missing variables, a more precise statement on the boundaries of generalisability, and an explicit acknowledgement of the potential for gestational age misclassification.
