# Supplementary material for: Early antenatal care initiation and associated factors in Hohoe Municipality, Ghana: A five-year multi-facility repeated cross-sectional record review study
Source: PLoS One. 2026 Nov 4;21(11):e0356427. doi: 10.1371/journal.pone.0356427 (PMC13734592; doi:10.1371/journal.pone.0356427)
Supplement: Supplementary file 3 [file pone.0356427.s003.docx]

**RESPONSE TO REVIEWERS’ COMMENTS**

Additional Editor Comments:

- Figure 2 can be removed and the information can be added to Table 1.

Response: We thank you for this suggestion. Information from figure 2 has been added to Table 1 accordingly.

- Please compare your study findings in discussion with other LMICs, such as Afghanistan, Ethiopia...

Response: We thank you for this suggestion. Studies from other LMICs have been included to the existing ones in the discussion

- In-text citation are not correctly used. Please review a few papers at PLOS ONE and correct in-text citations, particularly in discussion section. The in-text citation should come at the end of the sentence before the full stop.

Response: Thank you for this insight. Citations have been done accordingly

- Line 69: Please add the abbreviated form WHO after World Health Organization.

Response: Thank you for the suggestion. Abbreviation has been added accordingly.

Review Comments to the Author

Reviewer #1: Thank you so much dear Authors. Most of the comments were addressed appropriately. Dear authors, you can acknowledge AUC=0.58 and pseudor2=0.015 as your limitations.

Response: Thank you for the suggestion. AUC=0.58 and pseudor2=0.015 have been duly acknowledged in the limitations.
