# Supplementary material for: Early antenatal care initiation and associated factors in Hohoe Municipality, Ghana: A five-year multi-facility repeated cross-sectional record review study
Source: PLoS One. 2026 Nov 4;21(11):e0356427. doi: 10.1371/journal.pone.0356427 (PMC13734592; doi:10.1371/journal.pone.0356427)
Supplement: Supplementary file 4 [file pone.0356427.s004.docx]

**RESPONSE TO THE EDITOR**

- The Results section of the abstract reports OR, while the main results reports AOR. This should be consistent.

Response: Thank you for this valuable observation. We have revised the Results section of the abstract to ensure consistency with the main Results section. The reported odds ratios have been updated to adjusted odds ratios (aORs), consistent with the multivariable logistic regression analyses presented in the manuscript.

- The abstract mention that early ANC initiation increased slightly from 45.3% in 2020 to 46.4% in 2023. What about 2024? The study period is 2020-2024.

Response: Thank you for this observation. We have revised the abstract to include the 2024 data for completeness and consistency with the study period. The sentence now reads: “Early ANC initiation increased slightly from 45.3% in 2020 to 46.4% in 2023 and then declined to 44.3% in 2024, with an overall prevalence of 45.1% over the study period.” This accurately reflects the trend across the entire 2020–2024 study period.

- Major Point: The findings of interaction term are presented in the abstract and discussion. However, no such findings or Table is reported in main results section.

Response: Thank you for this observation. The interaction findings have now been formally reported in the main results section. A dedicated paragraph presenting the age-by-parity interaction model findings has been added, accompanied by a separate table (Table 5) displaying the full interaction model output, including all main effects, interaction terms, and model fit statistics. The two statistically significant interaction terms, women aged 31–40 who were multiparous (OR = 0.61; 95% CI: 0.41–0.91; p = 0.016) and those aged 31–40 who were grand multiparous (OR = 0.29; 95% CI: 0.11–0.77; p = 0.013) are clearly identified in the table and discussed in the results section. Page 22-23.

- Facilities and health centers are named differently across the document. Choose one terminology and use it consistently.

Response: Thank you for this valuable observation. We have reviewed the manuscript and ensured consistent terminology throughout. The official names of the individual study sites Gbi-Wegbe Health Center, Adabraka Health Center, and Volta Regional Hospital, have been retained where appropriate. This revision improves consistency while preserving the correct names of the participating facilities.

- What is the exact VIF? There is inconsistency between methods and strengths section.

Response: Thank you for pointing out this inconsistency. We have revised the manuscript to report the exact variance inflation factor (VIF) values consistently. Specifically, multicollinearity was assessed using variance inflation factors (VIFs), which ranged from 1.00 to 1.73, with a mean VIF of 1.31, indicating no evidence of problematic multicollinearity. This information is now reported consistently in the relevant sections of the manuscript.

- Line 323: “overall initiation rates did not differ significantly between the COVID-19 and post-COVID-19 periods...” However, the manuscript never reports an overall statistical comparison between the two periods.

Response: Thank you for this insightful comment. We agree that the manuscript did not report an overall statistical comparison between the COVID-19 and post-COVID-19 periods. Accordingly, we have revised the discussion by removing this unsupported statement and limiting our interpretation to the facility-specific trends that are supported by the reported analyses.
