# Supplementary material for: Early antenatal care initiation and associated factors in Hohoe Municipality, Ghana: A five-year multi-facility repeated cross-sectional record review study
Source: PLoS One. 2026 Nov 4;21(11):e0356427. doi: 10.1371/journal.pone.0356427 (PMC13734592; doi:10.1371/journal.pone.0356427)
Supplement: Supplementary file 5 [file pone.0356427.s005.docx]

**RESPONSE TO THE EDITOR**

Reviewer #4:

• The number of repeated participants excluded should be reported and specified in the study flow chart.

Response: The number of excluded transfer records has been reported in the methods section and the study flowchart updated. A total of 1,381 records were excluded 325 at Gbi-Wegbe Health Center, 274 at Adabraka Health Center, and 782 at Volta Regional Hospital leaving 6,279 records for the final analysis.

• The temporal trend should be assessed using logistic regression with survey year included as a predictor.

Response: Logistic regression models with survey year as both a continuous and categorical predictor have been fitted and reported in the results section. Survey year was non-significant in both the unadjusted (OR = 1.00; p = 0.926) and adjusted (OR = 1.02; p = 0.207) models, formally confirming the absence of a meaningful overall temporal trend.

• The facility-stratified analyses are of particular concern because fitting separate models does not formally test whether associations differ across facilities A pooled logistic regression model including facility-by-predictor interaction terms is recommended.

Response: Pooled logistic regression models incorporating facility-by-predictor interaction terms have been fitted and reported. The age-by-facility and parity-by-facility interactions were non-significant, formally confirming that these effects operate consistently across facilities. The hypertension-by-facility interaction identified one significant term: Grade 1 hypertension at Adabraka Health Center (OR = 3.90; 95% CI: 1.04–14.61; p = 0.044).

• For comparisons involving COVID-19 periods, logistic regression would be preferable to the chi-square test.

Response: The chi-square comparisons have been replaced with logistic regression. A pooled period-by-facility interaction model confirmed significant heterogeneity across facilities. The Volta Regional Hospital finding, previously non-significant by chi-square, was formally confirmed as significant (OR = 1.54; 95% CI: 1.17–2.02; p = 0.002). Table 2 has been updated accordingly.

• Lines 229–239 should therefore be moved to the Results section.

Response: The content from lines 229–239 has been moved to the Results section as recommended.
