# Supplementary material for: Early antenatal care initiation and associated factors in Hohoe Municipality, Ghana: A five-year multi-facility repeated cross-sectional record review study
Source: PLoS One. 2026 Nov 4;21(11):e0356427. doi: 10.1371/journal.pone.0356427 (PMC13734592; doi:10.1371/journal.pone.0356427)
Supplement: Peer review history

# Peer review: Decision Letter 0

**DOI:** 10.1371/journal.pone.0356427.r001

**Authors:** Muhammad Haroon Stanikzai

14 May 2026

Dear Dr. Delali,

Thank you for submitting your manuscript to PLOS ONE. After careful consideration, we feel that it has merit but does not fully meet PLOS ONE’s publication criteria as it currently stands. Therefore, we invite you to submit a revised version of the manuscript that addresses the points raised during the review process.

Please submit your revised manuscript by Jun 28 2026 11:59PM. If you will need more time than this to complete your revisions, please reply to this message or contact the journal office at plosone@plos.org. When you're ready to submit your revision, log on to https://www.editorialmanager.com/pone/ and select the 'Submissions Needing Revision' folder to locate your manuscript file.

- A letter that responds to each point raised by the academic editor and reviewer(s). You should upload this letter as a separate file labeled 'Response to Reviewers'.
- A marked-up copy of your manuscript that highlights changes made to the original version. You should upload this as a separate file labeled 'Revised Manuscript with Track Changes'.
- An unmarked version of your revised paper without tracked changes. You should upload this as a separate file labeled 'Manuscript'.

If you would like to make changes to your financial disclosure, please include your updated statement in your cover letter. Guidelines for resubmitting your figure files are available below the reviewer comments at the end of this letter.

If applicable, we recommend that you deposit your laboratory protocols in protocols.io to enhance the reproducibility of your results. Protocols.io assigns your protocol its own identifier (DOI) so that it can be cited independently in the future. For instructions see: https://journals.plos.org/plosone/s/submission-guidelines#loc-laboratory-protocols. Additionally, PLOS ONE offers an option for publishing peer-reviewed Lab Protocol articles, which describe protocols hosted on protocols.io. Read more information on sharing protocols at https://plos.org/protocols?utm_medium=editorial-email&utm_source=authorletters&utm_campaign=protocols.

As the corresponding author, your ORCID iD is verified in the submission system and will appear in the published article. PLOS supports the use of ORCID, and we encourage all coauthors to register for an ORCID iD and use it as well. Please encourage your coauthors to verify their ORCID iD within the submission system before final acceptance, as unverified ORCID iDs will not appear in the published article. *Only*  the individual author can complete the verification step; PLOS staff *cannot*  verify ORCID iDs on behalf of authors.

We look forward to receiving your revised manuscript.

Kind regards,

Muhammad Haroon Stanikzai

Academic Editor

PLOS One

Journal Requirements:

When submitting your revision, we need you to address these additional requirements.

1.Please ensure that your manuscript meets PLOS ONE's style requirements, including those for file naming. The PLOS ONE style templates can be found at

https://journals.plos.org/plosone/s/file?id=wjVg/PLOSOne_formatting_sample_main_body.pdf and

https://journals.plos.org/plosone/s/file?id=ba62/PLOSOne_formatting_sample_title_authors_affiliations.pdf

2. In the online submission form you indicate that your data is not available for proprietary reasons and have provided a contact point for accessing this data. Please note that your current contact point is a co-author on this manuscript. According to our Data Policy, the contact point must not be an author on the manuscript and must be an institutional contact, ideally not an individual. Please revise your data statement to a non-author institutional point of contact, such as a data access or ethics committee, and send this to us via return email. Please also include contact information for the third party organization, and please include the full citation of where the data can be found.

3. Your ethics statement should only appear in the Methods section of your manuscript. If your ethics statement is written in any section besides the Methods, please move it to the Methods section and delete it from any other section. Please ensure that your ethics statement is included in your manuscript, as the ethics statement entered into the online submission form will not be published alongside your manuscript.

4. We note that Figure 1 in your submission contain map images which may be copyrighted. All PLOS content is published under the Creative Commons Attribution License (CC BY 4.0), which means that the manuscript, images, and Supporting Information files will be freely available online, and any third party is permitted to access, download, copy, distribute, and use these materials in any way, even commercially, with proper attribution. For these reasons, we cannot publish previously copyrighted maps or satellite images created using proprietary data, such as Google software (Google Maps, Street View, and Earth). For more information, see our copyright guidelines: http://journals.plos.org/plosone/s/licenses-and-copyright.

We require you to either (1) present written permission from the copyright holder to publish these figures specifically under the CC BY 4.0 license, or (2) remove the figures from your submission:

1. You may seek permission from the original copyright holder of Figure 1 to publish the content specifically under the CC BY 4.0 license.

We recommend that you contact the original copyright holder with the Content Permission Form (http://journals.plos.org/plosone/s/file?id=7c09/content-permission-form.pdf) and the following text:

“I request permission for the open-access journal PLOS ONE to publish XXX under the Creative Commons Attribution License (CCAL) CC BY 4.0 (http://creativecommons.org/licenses/by/4.0/). Please be aware that this license allows unrestricted use and distribution, even commercially, by third parties. Please reply and provide explicit written permission to publish XXX under a CC BY license and complete the attached form.”

Please upload the completed Content Permission Form or other proof of granted permissions as an "Other" file with your submission.

In the figure caption of the copyrighted figure, please include the following text: “Reprinted from [ref] under a CC BY license, with permission from [name of publisher], original copyright [original copyright year].”

2. If you are unable to obtain permission from the original copyright holder to publish these figures under the CC BY 4.0 license or if the copyright holder’s requirements are incompatible with the CC BY 4.0 license, please either i) remove the figure or ii) supply a replacement figure that complies with the CC BY 4.0 license. Please check copyright information on all replacement figures and update the figure caption with source information. If applicable, please specify in the figure caption text when a figure is similar but not identical to the original image and is therefore for illustrative purposes only.

The following resources for replacing copyrighted map figures may be helpful:

USGS National Map Viewer (public domain): http://viewer.nationalmap.gov/viewer/

The Gateway to Astronaut Photography of Earth (public domain): http://eol.jsc.nasa.gov/sseop/clickmap/

Maps at the CIA (public domain): https://www.cia.gov/library/publications/the-world-factbook/index.html and https://www.cia.gov/library/publications/cia-maps-publications/index.html

NASA Earth Observatory (public domain): http://earthobservatory.nasa.gov/

Landsat: http://landsat.visibleearth.nasa.gov/

USGS EROS (Earth Resources Observatory and Science (EROS) Center) (public domain): http://eros.usgs.gov/#

Natural Earth (public domain): http://www.naturalearthdata.com/

5. If the reviewer comments include a recommendation to cite specific previously published works, please review and evaluate these publications to determine whether they are relevant and should be cited. There is no requirement to cite these works unless the editor has indicated otherwise.

Additional Editor Comments:

Three review reports have been obtained. Please revise your manuscript carefully considering esteemed reviewers' comments and suggestions.

[Note: HTML markup is below. Please do not edit.]

Reviewers' comments:

Reviewer's Responses to Questions

**Comments to the Author**

1. Is the manuscript technically sound, and do the data support the conclusions?

Reviewer #1: Partly

Reviewer #2: Partly

Reviewer #3: Yes

**********

2. Has the statistical analysis been performed appropriately and rigorously?

Reviewer #1: Yes

Reviewer #2: No

Reviewer #3: Yes

**********

3. Have the authors made all data underlying the findings in their manuscript fully available?

The PLOS Data policy

Reviewer #1: Yes

Reviewer #2: Yes

Reviewer #3: No

**********

4. Is the manuscript presented in an intelligible fashion and written in standard English?

Reviewer #1: No

Reviewer #2: Yes

Reviewer #3: Yes

**********

Reviewer #1: Dear Authors,

There are some suggestions to make your study stronger.

There is a incosistency between abstract and results section.

The introduction is too long, you can make it concise.

The discussion section is very good but it is better to discuss about your unexpected results (for example: hypertension and early ANC).

The implication of the study is too general, make it specific.

Conclusion is too general and you have to write based on your findings.

Reviewer #2: General assessment

Strengths

Relevant maternal/public health topic in sub-Saharan Africa.

Large retrospective dataset over 5 years.

Multi-facility design including primary and referral settings.

Use of adjusted logistic regression.

Trend component adds some value.

Major concerns

Novelty is limited

Similar studies already exist nationally in Ghana and regionally.

Authors must better justify why Hohoe municipality data adds substantial new evidence.

Question to authors:

What specific knowledge gap does this study fill beyond prior Ghana DHS analyses and nearby district studies?

TITLE AND ABSTRACT

Title (Lines 1–2)

Comment: Title is clear but somewhat descriptive and lengthy.

Suggested revision:

Early antenatal care initiation and associated factors in Hohoe Municipality, Ghana: a five-year retrospective facility-based study

Abstract

Lines 24–28 (Background)

Comment: Good rationale.

Question:

Why were only selected facilities included instead of all 21 facilities in Hohoe Municipality?

This may affect representativeness.

Lines 29–35 (Methods)

Comment: Methods are generally clear.

Concern: Authors mention retrospective analysis but not missing data handling.

Question:

How many records were excluded due to incompleteness or duplication before arriving at 6,279?

A flow diagram would improve transparency.

Lines 37–39 (Results)

Major issue: inconsistency

Abstract says:

2020 = 44.3%

2024 = 46.4%

But Results section Figure 3 states:

2020 = 45.3%

2024 = 44.3%

These are inconsistent.

Question to authors:

Please reconcile discrepancies in yearly prevalence values between abstract and Results/Figure 3.

This is a serious reporting issue.

Lines 40–51

Comment: Too many adjusted ORs in abstract.

Recommendation:

Reduce detailed estimates and retain only strongest predictors.

Lines 53–58 (Conclusion)

Concern: “Reducing high parity” is not an immediate intervention target.

Parity is a reproductive history variable, not easily modifiable.

Suggested wording:

“…targeted interventions focusing on reproductive counseling, early pregnancy recognition, and anemia prevention…”

INTRODUCTION / BACKGROUND

Lines 62–83

Comment: Background is comprehensive but verbose.

Could be shortened by ~20%.

Examples:

maternal mortality discussion is overly long for a local facility study.

Lines 79–80

Issue: suspicious estimate

Authors report:

“0.2% early initiation in Niger to 16.6% in Liberia”

These seem implausibly low.

Question:

Please verify these estimates and citations.

Potential citation or interpretation error.

Lines 99–113

Comment: This is where novelty should be strengthened.

Currently:

“Hohoe lacks retrospective analysis”

This alone is weak.

Authors should explicitly argue:

post-COVID period,

facility-level trends,

clinical predictors unavailable in DHS.

METHODS

Study design (Lines 116–125)

Line 117

Comment: “retrospective cross-sectional” acceptable.

But because 5-year records analyzed:

Could also be described as repeated cross-sectional record review.

Line 124

Question:

Please provide STROBE checklist as supplementary file.

Study site (126–139)

Too detailed.

Geographical boundaries can be condensed.

No need for long municipal description.

Inclusion/exclusion (147–163)

Major missing information

Authors say incomplete records excluded.

Question:

How many records were excluded for:

missing gestational age,

transfer-in cases,

duplicates?

Need explicit numbers.

Sample size (164–170)

Fine.

But authors should clarify denominator:

Were all first ANC attendees included only?

Study variables (171–195)

Major methodological concern

Lines 179–184

Authors include:

parity

gravidity

Both are highly correlated conceptually.

Even though VIF is low, this may introduce conceptual redundancy.

Question:

Why were parity and gravidity included simultaneously in the same regression model?

This may complicate interpretation.

Line 183–184

Problem:

“We coded gravida into 0 = primigravida and 1 = multigravida.”

This is not ideal wording.

Use:

primigravida vs multigravida.

Lines 187–193

Blood pressure categories included.

But causal interpretation is problematic.

BP measured at first visit is concurrent with outcome timing.

Thus hypertension cannot truly predict early ANC.

It is measured after care-seeking.

Major concern

Question:

How do authors justify interpreting blood pressure as a determinant rather than a concurrent clinical characteristic?

Same issue applies to hemoglobin.

Sampling/data collection (196–219)

Well described.

But “representative” claim is too strong since facilities purposively selected.

Suggested revision:

Replace “representative” with “captures diverse care settings.”

Validity (220–232)

Line 230

“maximum agreement”

Not scientific wording.

Need kappa or percent agreement.

Question:

What was inter-rater reliability percentage?

DATA ANALYSIS

Lines 233–248

Major issue

Bivariate selection based on significance only.

This is not ideal.

Variables should be selected based on theory/literature.

Question:

Were all clinically relevant covariates included regardless of bivariate significance?

Missing model diagnostics

Need:

goodness of fit (Hosmer-Lemeshow or equivalent)

ROC/AUC maybe optional.

RESULTS

Figure 2

Typo:

“Late Eearly”

Needs correction.

Figure 3

Trend labels inconsistent with abstract.

Must correct.

Figure 5

Major concern

Facility-specific yearly proportions appear extreme:

2020:

Gbi-Wegbe = 11%

Adabraka = 5.5%

Volta Regional = 83.5%

This is highly unusual.

Could indicate denominator/coding issue.

Question:

Please explain why Volta Regional Hospital had 83.5% early initiation in 2020 while other facilities were <11%.

Potential data anomaly.

able 2

Major interpretation issue

Authors state:

multigravida more likely early (aOR 1.67)

higher parity less likely early

This seems contradictory.

Because gravidity and parity are related.

Likely model instability due to collinearity/conceptual overlap.

Question:

Did authors test model excluding either parity or gravidity?

Highly recommended.

DISCUSSION

Lines 375–381

Interpretation of multigravida contradicts parity discussion.

Needs reconciliation.

Authors currently say:

prior experience delays care

AND

prior experience improves early care.

Both cannot be simultaneously generalized without nuance.

Need clearer explanation.

Lines 383–391

Problematic causal language.

Authors imply anemia influences ANC timing.

Actually reverse may be possible:

late ANC may increase probability of anemia detection later.

Recommendation: Reframe as association only.

CONCLUSION

Lines 449–461

Issue:

Recommendations exceed study evidence.

Example:

mobile ANC clinics not directly studied.

Recommendation should align with findings.

Suggested:

targeted maternal education,

improved facility experience,

screening awareness.

ABBREVIATIONS

Line 465

Error:

MoH incorrectly expanded as:

Ministry of Finance

Should be:

Ministry of Health

REFERENCES

Need formatting consistency check:

capitalization inconsistent,

journal styles inconsistent.

Reviewer #3: This manuscript reports a retrospective, multi-facility, cross-sectional study examining the prevalence, annual trends, and determinants of early antenatal care (ANC) initiation among 6,279 pregnant women attending three public health facilities in Hohoe Municipality, Ghana, between January 2020 and December 2024.

Comment 1

The abstract states that early ANC initiation “slightly increased from 44.3% in 2020 to 46.4% in 2024”. However, Figure 3 and the accompanying results text show the opposite direction: 2020 started at 45.3%, dipped to 44.7%-44.8% in 2021-2022, rose to a peak of 46.4% in 2023, and then fell back to 44.3% in 2024. The 2024 figure of 44.3% is therefore lower than the 2020 figure of 45.3%, and the peak year was 2023, not 2024.

Comment 2

Figure 5 reports that Volta Regional Hospital had an early ANC initiation rate of 83.5% in 2020, which then declined sharply to 44.7% by 2024. At the same time, Figure 4 reports the five-year pooled early initiation rate at Volta Regional Hospital as 46.4%, and Gbi-Wegbe Health Center as 48.4%. If Volta Regional Hospital began at 83.5% in 2020, the pooled five-year figure of 46.4% implies very low rates in subsequent years to average down to that level. Yet Gbi-Wegbe's pooled rate (48.4%) exceeds Volta's (46.4%), despite the latter starting at 83.5%. The manuscript must reconcile these figures explicitly, including the number of records contributed by each facility per year, and provide a narrative explanation for the extreme decline at Volta Regional Hospital.

Comment 3

The manuscript includes both gravidity (primigravida vs. multigravida) and parity (nullipara, primipara, multipara, grand multipara) as independent predictors in the same multivariable model. These two variables are conceptually and empirically highly correlated: a primigravida woman is by definition nulliparous, and multigravida women are almost always parous. Despite the authors' VIF assessment showing values below 2.42, VIF is not sensitive to perfect or near-perfect category-level collinearity when variables are categorical. The crude odds ratio for gravidity is 1.04 (not significant, p=0.482), yet the adjusted OR is 1.67 (p<0.001), which represents a large and statistically significant shift that is unusual and may reflect collinearity-driven instability rather than a genuine independent effect. The authors must provide a conceptual and statistical justification for including both variables simultaneously.

Comment 4

The abstract reports the adjusted OR for moderate anaemia as aOR=0.25 (95% CI: 0.22–0.39). However, Table 2 reports this as aOR=0.25 (95% CI: 0.22–0.29). These two confidence intervals have the same lower bound (0.22) but different upper bounds (0.39 in the abstract vs. 0.29 in the table). The correct value must be identified from the Stata output and the discrepancy corrected.

The following issues should be corrected to improve accuracy and clarity.

1. The figure legend for Figure 2 (the pie chart) labels the early ANC slice as "Eearly" — this is a typographical error and should read "Early".

2. The list of abbreviations defines MoH as "Ministry of Finance" rather than "Ministry of Health". This should be corrected.

3. The manuscript describes the data availability as follows: "The datasets used or analysed during the current study are available from the corresponding author on reasonable request." PLOS ONE requires that underlying data be fully publicly available without restriction, not merely available on request. The authors should either deposit the dataset in a public repository and provide the DOI, or provide a specific justification for why this is not feasible (for example, patient confidentiality concerns under Ghanaian data protection law), as PLOS ONE permits exceptions in documented circumstances.

4. The introduction contains the statement that “43% of pregnant women begin their first antenatal care visit late” globally, citing Reference 5 (Girma et al., 2023, an Addis Ababa facility-based study). A single-city facility study is not an appropriate source for a global statistic. A global or systematic review source should be substituted for this specific claim.

5. The conclusion calls for efforts to focus on "reducing high parity" as an intervention. Parity is not an intervenable exposure in the short term; the intended recommendation appears to be family planning promotion or birth spacing. The conclusion should be revised to reflect a realistic and ethically appropriate intervention target.

6. Reference 3 is listed as a UNFPA Turkey webpage, cited to support the statistic that 70% of maternal deaths occur in sub-Saharan Africa. A peer-reviewed source or the primary WHO/UNICEF/UNFPA report should be used instead of a country office webpage.

7. The discussion notes that the Volta Regional Hospital decline from 83.5% (2020) to 44.7% (2024) is presented without any substantive discussion of possible causes, despite being the most striking finding in the trend data. Given that this facility contributes over half of the total study sample (3,224 of 6,279 records), its trajectory dominates the pooled figures. A dedicated discussion of plausible explanations is essential for readers to interpret the overall trend correctly.

8. The study period includes the peak COVID-19 years of 2020 and 2021. While the background section discusses COVID-19's impact on ANC in Ghana, the results section makes no attempt to examine whether 2020-2021 patterns differ from 2022-2024, nor is this addressed in the discussion as a limitation on trend interpretation. A brief explicit comment on this is warranted.

The limitations section is present but should be expanded to acknowledge the following:

• The study relies exclusively on variables available in routine ANC registers. Key established predictors of early ANC initiation including education level, marital status, socioeconomic status, distance to facility, employment, and cultural or religious beliefs are entirely absent from the analysis. This is acknowledged briefly but should be foregrounded more prominently, as the regression model explains only part of the variation in early initiation, and the missing variables are among those most consistently associated with ANC timing in the broader literature.

• The purposive selection of three facilities out of twenty-one in the municipality means that the majority of primary care facilities are not represented. The authors argue that the selected facilities provide a spectrum of care, which is reasonable, but the claim that findings are "representative of ANC patterns across the municipality" is overstated given that 18 facilities are excluded. The limitations section should clarify the boundaries of generalisability more precisely.

• The retrospective record-review design means that gestational age at first visit was recorded as a clinical estimate, not confirmed by first-trimester ultrasound. In many resource-limited settings, gestational age is estimated from the last menstrual period, which is subject to recall bias and irregular cycle variation. The potential for misclassification of early versus late initiation, the primary outcome, should be acknowledged.

**********

what does this mean?). If published, this will include your full peer review and any attached files.

If you choose “no”, your identity will remain anonymous but your review may still be made public.

**Do you want your identity to be public for this peer review?**  For information about this choice, including consent withdrawal, please see our Privacy Policy

Reviewer #1: No

Reviewer #2: No

Reviewer #3: **Yes:**  Ritah Nantale

**********

[NOTE: If reviewer comments were submitted as an attachment file, they will be attached to this email and accessible via the submission site. Please log into your account, locate the manuscript record, and check for the action link "View Attachments". If this link does not appear, there are no attachment files.]

To ensure your figures meet our technical requirements, please review our figure guidelines: https://journals.plos.org/plosone/s/figures

You may also use PLOS’s free figure tool, NAAS, to help you prepare publication quality figures: https://journals.plos.org/plosone/s/figures#loc-tools-for-figure-preparation.

NAAS will assess whether your figures meet our technical requirements by comparing each figure against our figure specifications.

# Author comment: Author response to Decision Letter 1

**DOI:** 10.1371/journal.pone.0356427.r002

1 Jun 2026

Have no response to provide. All responses to comment are within the "Response to Reviewers" file.

Attachment Submitted filename: Response to Reviewers.docx

# Peer review: Decision Letter 1

**DOI:** 10.1371/journal.pone.0356427.r003

**Authors:** Muhammad Haroon Stanikzai

8 Jun 2026

Dear Dr. Delali,

Thank you for submitting your manuscript to PLOS ONE. After careful consideration, we feel that it has merit but does not fully meet PLOS ONE’s publication criteria as it currently stands. Therefore, we invite you to submit a revised version of the manuscript that addresses the points raised during the review process.

==============================

The reviewers requested a minor revision and some editing corrections need to be done detailed at additional editor comments section.

==============================

Please submit your revised manuscript by Jul 23 2026 11:59PM. If you will need more time than this to complete your revisions, please reply to this message or contact the journal office at plosone@plos.org. When you're ready to submit your revision, log on to https://www.editorialmanager.com/pone/ and select the 'Submissions Needing Revision' folder to locate your manuscript file.

- A letter that responds to each point raised by the academic editor and reviewer(s). You should upload this letter as a separate file labeled 'Response to Reviewers'.
- A marked-up copy of your manuscript that highlights changes made to the original version. You should upload this as a separate file labeled 'Revised Manuscript with Track Changes'.
- An unmarked version of your revised paper without tracked changes. You should upload this as a separate file labeled 'Manuscript'.

If you would like to make changes to your financial disclosure, please include your updated statement in your cover letter. Guidelines for resubmitting your figure files are available below the reviewer comments at the end of this letter.

If applicable, we recommend that you deposit your laboratory protocols in protocols.io to enhance the reproducibility of your results. Protocols.io assigns your protocol its own identifier (DOI) so that it can be cited independently in the future. For instructions see: https://journals.plos.org/plosone/s/submission-guidelines#loc-laboratory-protocols. Additionally, PLOS ONE offers an option for publishing peer-reviewed Lab Protocol articles, which describe protocols hosted on protocols.io. Read more information on sharing protocols at https://plos.org/protocols?utm_medium=editorial-email&utm_source=authorletters&utm_campaign=protocols.

As the corresponding author, your ORCID iD is verified in the submission system and will appear in the published article. PLOS supports the use of ORCID, and we encourage all coauthors to register for an ORCID iD and use it as well. Please encourage your coauthors to verify their ORCID iD within the submission system before final acceptance, as unverified ORCID iDs will not appear in the published article. *Only*  the individual author can complete the verification step; PLOS staff *cannot*  verify ORCID iDs on behalf of authors.

We look forward to receiving your revised manuscript.

Kind regards,

Muhammad Haroon Stanikzai

Academic Editor

PLOS One

Journal Requirements:

If the reviewer comments include a recommendation to cite specific previously published works, please review and evaluate these publications to determine whether they are relevant and should be cited. There is no requirement to cite these works unless the editor has indicated otherwise.

Please review your reference list to ensure that it is complete and correct. If you have cited papers that have been retracted, please include the rationale for doing so in the manuscript text, or remove these references and replace them with relevant current references. Any changes to the reference list should be mentioned in the rebuttal letter that accompanies your revised manuscript. If you need to cite a retracted article, indicate the article’s retracted status in the References list and also include a citation and full reference for the retraction notice.

Additional Editor Comments:

- Figure 2 can be removed and the information can be added to Table 1.

- Please compare your study findings in discussion with other LMICs, such as Afghanistan, Ethiopia...

- In-text citation are not correctly used. Please review a few papers at PLOS ONE and correct in-text citations, particularly in discussion section. The in-text citation should come at the end of the sentence before the full stop.

- Line 69: Please add the abbreviated form WHO after World Health Organization.

[Note: HTML markup is below. Please do not edit.]

Reviewers' comments:

Reviewer's Responses to Questions

**Comments to the Author**

Reviewer #1: All comments have been addressed

Reviewer #2: All comments have been addressed

**********

2. Is the manuscript technically sound, and do the data support the conclusions?

Reviewer #1: Partly

Reviewer #2: Yes

**********

3. Has the statistical analysis been performed appropriately and rigorously?

Reviewer #1: Yes

Reviewer #2: Yes

**********

4. Have the authors made all data underlying the findings in their manuscript fully available?

The PLOS Data policy

Reviewer #1: Yes

Reviewer #2: Yes

**********

5. Is the manuscript presented in an intelligible fashion and written in standard English?

Reviewer #1: No

Reviewer #2: Yes

**********

Reviewer #1: Thank you so much dear Authors. Most of the comments were addressed appropriately. Dear authors, you can acknowledge AUC=0.58 and pseudor2=0.015 as your limitations.

Reviewer #2: This revision is now publication ready, since it addressed all my concerns during the previous interactions

**********

what does this mean?). If published, this will include your full peer review and any attached files.

If you choose “no”, your identity will remain anonymous but your review may still be made public.

**Do you want your identity to be public for this peer review?**  For information about this choice, including consent withdrawal, please see our Privacy Policy

Reviewer #1: No

Reviewer #2: No

**********

[NOTE: If reviewer comments were submitted as an attachment file, they will be attached to this email and accessible via the submission site. Please log into your account, locate the manuscript record, and check for the action link "View Attachments". If this link does not appear, there are no attachment files.]

To ensure your figures meet our technical requirements, please review our figure guidelines: https://journals.plos.org/plosone/s/figures

You may also use PLOS’s free figure tool, NAAS, to help you prepare publication quality figures: https://journals.plos.org/plosone/s/figures#loc-tools-for-figure-preparation.

NAAS will assess whether your figures meet our technical requirements by comparing each figure against our figure specifications.

# Author comment: Author response to Decision Letter 2

**DOI:** 10.1371/journal.pone.0356427.r004

11 Jun 2026

None

Attachment Submitted filename: Response_to_Reviewers_auresp_2.docx

# Editor report: Decision Letter 2

**DOI:** 10.1371/journal.pone.0356427.r005

**Authors:** Muhammad Haroon Stanikzai

1 Jul 2026

Dear Dr. Delali,

Thank you for submitting your manuscript to PLOS ONE. After careful consideration, we feel that it has merit but does not fully meet PLOS ONE’s publication criteria as it currently stands. Therefore, we invite you to submit a revised version of the manuscript that addresses the points raised during the review process.

Please submit your revised manuscript by Aug 15 2026 11:59PM. If you will need more time than this to complete your revisions, please reply to this message or contact the journal office at plosone@plos.org. When you're ready to submit your revision, log on to https://www.editorialmanager.com/pone/ and select the 'Submissions Needing Revision' folder to locate your manuscript file.

- A letter that responds to each point raised by the academic editor and reviewer(s). You should upload this letter as a separate file labeled 'Response to Reviewers'.
- A marked-up copy of your manuscript that highlights changes made to the original version. You should upload this as a separate file labeled 'Revised Manuscript with Track Changes'.
- An unmarked version of your revised paper without tracked changes. You should upload this as a separate file labeled 'Manuscript'.

If you would like to make changes to your financial disclosure, please include your updated statement in your cover letter. Guidelines for resubmitting your figure files are available below the reviewer comments at the end of this letter.

If applicable, we recommend that you deposit your laboratory protocols in protocols.io to enhance the reproducibility of your results. Protocols.io assigns your protocol its own identifier (DOI) so that it can be cited independently in the future. For instructions see: https://journals.plos.org/plosone/s/submission-guidelines#loc-laboratory-protocols. Additionally, PLOS ONE offers an option for publishing peer-reviewed Lab Protocol articles, which describe protocols hosted on protocols.io. Read more information on sharing protocols at https://plos.org/protocols?utm_medium=editorial-email&utm_source=authorletters&utm_campaign=protocols.

As the corresponding author, your ORCID iD is verified in the submission system and will appear in the published article. PLOS supports the use of ORCID, and we encourage all coauthors to register for an ORCID iD and use it as well. Please encourage your coauthors to verify their ORCID iD within the submission system before final acceptance, as unverified ORCID iDs will not appear in the published article. *Only*  the individual author can complete the verification step; PLOS staff *cannot*  verify ORCID iDs on behalf of authors.

We look forward to receiving your revised manuscript.

Kind regards,

Muhammad Haroon Stanikzai

Academic Editor

PLOS One

Journal Requirements:

If the reviewer comments include a recommendation to cite specific previously published works, please review and evaluate these publications to determine whether they are relevant and should be cited. There is no requirement to cite these works unless the editor has indicated otherwise.

Please review your reference list to ensure that it is complete and correct. If you have cited papers that have been retracted, please include the rationale for doing so in the manuscript text, or remove these references and replace them with relevant current references. Any changes to the reference list should be mentioned in the rebuttal letter that accompanies your revised manuscript. If you need to cite a retracted article, indicate the article’s retracted status in the References list and also include a citation and full reference for the retraction notice.

Additional Editor Comments:

- The Results section of the abstract reports OR, while the main results reports AOR. This should be consistent.

- The abstract mention that early ANC initiation increased slightly from 45.3% in 2020 to 46.4% in 2023. What about 2024? The study period is 2020-2024.

- Major Point: The findings of interaction term are presented in the abstract and discussion. However, no such findings or Table is reported in main results section.

- Facilities and health centers are named differently across the document. Choose one terminology and use it consistently.

- What is the exact VIF? There is inconsistency between methods and strengths section.

- Line 323: "overall initiation rates did not differ significantly between the COVID-19 and post-COVID-19 periods..." However, the manuscript never reports an overall statistical comparison between the two periods.

[Note: HTML markup is below. Please do not edit.]

[NOTE: If reviewer comments were submitted as an attachment file, they will be attached to this email and accessible via the submission site. Please log into your account, locate the manuscript record, and check for the action link "View Attachments". If this link does not appear, there are no attachment files.]

To ensure your figures meet our technical requirements, please review our figure guidelines: https://journals.plos.org/plosone/s/figures

You may also use PLOS’s free figure tool, NAAS, to help you prepare publication quality figures: https://journals.plos.org/plosone/s/figures#loc-tools-for-figure-preparation.

NAAS will assess whether your figures meet our technical requirements by comparing each figure against our figure specifications.

# Author comment: Author response to Decision Letter 3

**DOI:** 10.1371/journal.pone.0356427.r006

13 Jul 2026

Responses are within the uploaded document labeled 'Response to Reviewers'.

Attachment Submitted filename: Response_to_Reviewers_auresp_3.docx

# Peer review: Decision Letter 3

**DOI:** 10.1371/journal.pone.0356427.r007

**Authors:** Helen Howard

11 Sep 2026

Early antenatal care initiation and associated factors in Hohoe Municipality, Ghana: a five-year multi-facility repeated cross-sectional record review study

PLOS One

Dear Dr. Delali,

Thank you for submitting your manuscript to PLOS One. After careful consideration, we feel that it has merit but does not fully meet PLOS One’s publication criteria as it currently stands. Therefore, we invite you to submit a revised version of the manuscript that addresses the points raised during the review process.

Please submit your revised manuscript by Oct 25 2026 11:59PM. If you will need more time than this to complete your revisions, please reply to this message or contact the journal office at plosone@plos.org. When you're ready to submit your revision, log on to https://www.editorialmanager.com/pone/ and select the 'Submissions Needing Revision' folder to locate your manuscript file.

Please include the following items when submitting your revised manuscript:

- A letter that responds to each point raised by the academic editor and reviewer(s). You should upload this letter as a separate file labeled 'Response to Reviewers'.
- A marked-up copy of your manuscript that highlights changes made to the original version. You should upload this as a separate file labeled 'Revised Manuscript with Track Changes'.
- An unmarked version of your revised paper without tracked changes. You should upload this as a separate file labeled 'Manuscript'.

If you would like to make changes to your financial disclosure, please include your updated statement in your cover letter. Guidelines for resubmitting your figure files are available below the reviewer comments at the end of this letter.

If applicable, we recommend that you deposit your laboratory protocols in protocols.io to enhance the reproducibility of your results. Protocols.io assigns your protocol its own identifier (DOI) so that it can be cited independently in the future. For instructions see: https://journals.plos.org/plosone/s/submission-guidelines#loc-laboratory-protocols. Additionally, PLOS One offers an option for publishing peer-reviewed Lab Protocol articles, which describe protocols hosted on protocols.io. Read more information on sharing protocols at https://plos.org/protocols?utm_medium=editorial-email&utm_source=authorletters&utm_campaign=protocols.

As the corresponding author, your ORCID iD is verified in the submission system and will appear in the published article. PLOS supports the use of ORCID, and we encourage all coauthors to register for an ORCID iD and use it as well. Please encourage your coauthors to verify their ORCID iD within the submission system before final acceptance, as unverified ORCID iDs will not appear in the published article. *Only*  the individual author can complete the verification step; PLOS staff *cannot*  verify ORCID iDs on behalf of authors.

We look forward to receiving your revised manuscript.

Kind regards,

Helen Howard

Staff Editor

PLOS One

Journal Requirements:

1. If the reviewer comments include a recommendation to cite specific previously published works, please review and evaluate these publications to determine whether they are relevant and should be cited. There is no requirement to cite these works unless the editor has indicated otherwise.

[Note: HTML markup is below. Please do not edit.]

Reviewer's Responses to Questions

**Comments to the Author**

Reviewer #4: (No Response)

**********

2. Is the manuscript technically sound, and do the data support the conclusions?

Reviewer #4: (No Response)

**********

3. Has the statistical analysis been performed appropriately and rigorously?

Reviewer #4: (No Response)

**********

4. Have the authors made all data underlying the findings in their manuscript fully available?

The PLOS Data policy

Reviewer #4: (No Response)

**********

5. Is the manuscript presented in an intelligible fashion and written in standard English?

Reviewer #4: (No Response)

**********

Reviewer #4: • The number of repeated participants excluded should be reported and specified in the study flow chart.

• The temporal trend should be assessed using logistic regression with survey year included as a predictor.

• The facility-stratified analyses are of particular concern because fitting separate models does not formally test whether associations differ across facilities A pooled logistic regression model including facility-by-predictor interaction terms is recommended.

• For comparisons involving COVID-19 periods, logistic regression would be preferable to the chi-square test.

• Lines 229–239 should therefore be moved to the Results section.

**********

what does this mean?). If published, this will include your full peer review and any attached files.

If you choose “no”, your identity will remain anonymous but your review may still be made public.

**Do you want your identity to be public for this peer review?**  For information about this choice, including consent withdrawal, please see our Privacy Policy

Reviewer #4: No

**********

[NOTE: If reviewer comments were submitted as an attachment file, they will be attached to this email and accessible via the submission site. Please log into your account, locate the manuscript record, and check for the action link "View Attachments". If this link does not appear, there are no attachment files.]

To ensure your figures meet our technical requirements, please review our figure guidelines: https://journals.plos.org/plosone/s/figures

You may also use PLOS’s free figure tool, NAAS, to help you prepare publication quality figures: https://journals.plos.org/plosone/s/figures#loc-tools-for-figure-preparation.

NAAS will assess whether your figures meet our technical requirements by comparing each figure against our figure specifications.

# Author comment: Author response to Decision Letter 4

**DOI:** 10.1371/journal.pone.0356427.r008

17 Sep 2026

Review response document has been attached

Attachment Submitted filename: Response_to_Reviewers_auresp_4.docx

# Peer review: Decision Letter 4

**DOI:** 10.1371/journal.pone.0356427.r009

**Authors:** Emma Campbell

11 Oct 2026

Early antenatal care initiation and associated factors in Hohoe Municipality, Ghana: a five-year multi-facility repeated cross-sectional record review study

PONE-D-26-17280R4

Dear Dr. Delali,

We’re pleased to inform you that your manuscript has been judged scientifically suitable for publication and will be formally accepted for publication once it meets all outstanding technical requirements.

Within one week, you’ll receive an e-mail detailing the required amendments. When these have been addressed, you’ll receive a formal acceptance letter and your manuscript will be scheduled for publication.

An invoice will be generated when your article is formally accepted. Please note, if your institution has a publishing partnership with PLOS and your article meets the relevant criteria, all or part of your publication costs will be covered. Please make sure your user information is up-to-date by logging into Editorial Manager at Editorial Manager® and clicking the ‘Update My Information' link at the top of the page. For questions related to billing, please contact billing support.

If your institution or institutions have a press office, please notify them about your upcoming paper to help maximize its impact. If they’ll be preparing press materials, please inform our press team as soon as possible -- no later than 48 hours after receiving the formal acceptance. Your manuscript will remain under strict press embargo until 2 pm Eastern Time on the date of publication. For more information, please contact onepress@plos.org.

Kind regards,

Emma Campbell, Ph.D

Staff Editor

PLOS One

Additional Editor Comments (optional):

Reviewers' comments:

Reviewer's Responses to Questions

**Comments to the Author**

Reviewer #4: All comments have been addressed

**********

2. Is the manuscript technically sound, and do the data support the conclusions?

Reviewer #4: (No Response)

**********

3. Has the statistical analysis been performed appropriately and rigorously?

Reviewer #4: (No Response)

**********

4. Have the authors made all data underlying the findings in their manuscript fully available?

The PLOS Data policy

Reviewer #4: (No Response)

**********

5. Is the manuscript presented in an intelligible fashion and written in standard English?

Reviewer #4: (No Response)

**********

Reviewer #4: (No Response)

**********

what does this mean?). If published, this will include your full peer review and any attached files.

If you choose “no”, your identity will remain anonymous but your review may still be made public.

**Do you want your identity to be public for this peer review?**  For information about this choice, including consent withdrawal, please see our Privacy Policy

Reviewer #4: No

**********

# Editor report: Acceptance letter

**DOI:** 10.1371/journal.pone.0356427.r010

**Authors:** Emma Campbell

PONE-D-26-17280R4

PLOS One

Dear Dr. Delali,

I'm pleased to inform you that your manuscript has been deemed suitable for publication in PLOS One. Congratulations! Your manuscript is now being handed over to our production team.

At this stage, our production department will prepare your paper for publication. This includes ensuring the following:

* All references, tables, and figures are properly cited

* All relevant supporting information is included in the manuscript submission,

* There are no issues that prevent the paper from being properly typeset

You will receive further instructions from the production team, including instructions on how to review your proof when it is ready. Please keep in mind that we are working through a large volume of accepted articles, so please give us a few days to review your paper and let you know the next and final steps.

Lastly, if your institution or institutions have a press office, please let them know about your upcoming paper now to help maximize its impact. If they'll be preparing press materials, please inform our press team within the next 48 hours. Your manuscript will remain under strict press embargo until 2 pm Eastern Time on the date of publication. For more information, please contact onepress@plos.org.

You will receive an invoice from PLOS for your publication fee after your manuscript has reached the completed accept phase. If you receive an email requesting payment before acceptance or for any other service, this may be a phishing scheme. Learn how to identify phishing emails and protect your accounts at https://explore.plos.org/phishing.

If we can help with anything else, please email us at customercare@plos.org.

Thank you for submitting your work to PLOS One and supporting open access.

Kind regards,

PLOS One Editorial Office Staff

on behalf of

Dr Emma Campbell

Staff Editor

PLOS One
